# Respiratory support in patients with severe COVID-19 in the International Severe Acute Respiratory and Emerging Infection (ISARIC) COVID-19 study: a prospective, multinational, observational study

**DOI:** 10.1186/s13054-022-04155-1

**Published:** 2022-09-13

**Authors:** Luis Felipe Reyes, Srinivas Murthy, Esteban Garcia-Gallo, Laura Merson, Elsa D. Ibáñez-Prada, Jordi Rello, Yuli V. Fuentes, Ignacio Martin-Loeches, Fernando Bozza, Sara Duque, Fabio S. Taccone, Robert A. Fowler, Christiana Kartsonaki, Bronner P. Gonçalves, Barbara Wanjiru Citarella, Diptesh Aryal, Erlina Burhan, Matthew J. Cummings, Christelle Delmas, Rodrigo Diaz, Claudia Figueiredo-Mello, Madiha Hashmi, Prasan Kumar Panda, Miguel Pedrera Jiménez, Diego Fernando Bautista Rincon, David Thomson, Alistair Nichol, John C. Marshall, Piero L. Olliaro, the ISARIC Characterization Group, Ali Abbas, Sheryl Ann Abdukahil, Ryuzo Abe, Laurent Abel, Lara Absil, Subhash Acharya, Andrew Acker, Diana Adrião, Saleh Al Ageel, Shakeel Ahmed, Kate Ainscough, Tharwat Aisa, Ali Ait Hssain, Younes Ait Tamlihat, Takako Akimoto, Ernita Akmal, Eman Al Qasim, Razi Alalqam, Tala Al-dabbous, Senthilkumar Alegesan, Cynthia Alegre, Marta Alessi, Beatrice Alex, Kévin Alexandre, Abdulrahman Al-Fares, Huda Alfoudri, Imran Ali, Naseem Ali Shah, Kazali Enagnon Alidjnou, Jeffrey Aliudin, Qabas Alkhafajee, Clotilde Allavena, Nathalie Allou, Aneela Altaf, João Alves, João Melo Alves, Rita Alves, Joana Alves Cabrita, Maria Amaral, Phoebe Ampaw, Roberto Andini, Claire Andréjak, Andrea Angheben, François Angoulvant, Séverine Ansart, Massimo Antonelli, Carlos Alexandre Antunes de Brito, Ardiyan Apriyana, Yaseen Arabi, Irene Aragao, Carolline Araujo, Antonio Arcadipane, Patrick Archambault, Lukas Arenz, Jean-Benoît Arlet, Christel Arnold-Day, Lovkesh Arora, Rakesh Arora, Elise Artaud-Macari, Diptesh Aryal, Angel Asensio, Namra Asif, Mohammad Asim, Jean Baptiste Assie, Anika Atique, A. M. Udara Lakshan Attanyake, Johann Auchabie, Hugues Aumaitre, Adrien Auvet, Laurène Azemar, Cecile Azoulay, Benjamin Bach, Delphine Bachelet, Claudine Badr, Nadia Baig, J. Kenneth Baillie, Erica Bak, Agamemnon Bakakos, Andriy Bal, Valeria Balan, Firouzé Bani-Sadr, Renata Barbalho, Wendy S. Barclay, Michaela Barnikel, Helena Barrasa, Audrey Barrelet, Cleide Barrigoto, Marie Bartoli, Joaquín Baruch, Romain Basmaci, Denise Battaglini, Jules Bauer, Denisse Bazan Dow, Abigail Beane, Alexandra Bedossa, Husna Begum, Sylvie Behilill, Albertus Beishuizen, Aleksandr Beljantsev, David Bellemare, Anna Beltrame, Marine Beluze, Nicolas Benech, Dehbia Benkerrou, Suzanne Bennett, Luís Bento, Jan-Erik Berdal, Delphine Bergeaud, Hazel Bergin, José Luis Bernal Sobrino, Giulia Bertoli, Lorenzo Bertolino, Simon Bessis, Sybille Bevilcaqua, Karine Bezulier, Amar Bhatt, Krishna Bhavsar, Claudia Bianco, Moirangthem Bikram Singh, Felwa Bin Humaid, François Bissuel, Laurent Bitker, Jonathan Bitton, Pablo Blanco-Schweizer, Catherine Blier, Frank Bloos, Mathieu Blot, Filomena Boccia, Laetitia Bodenes, Debby Bogaert, Anne-Hélène Boivin, Pierre-Adrien Bolze, François Bompart, Diogo Borges, Raphaël Borie, Hans Martin Bosse, Elisabeth Botelho-Nevers, Lila Bouadma, Olivier Bouchaud, Sabelline Bouchez, Dounia Bouhmani, Damien Bouhour, Kévin Bouiller, Laurence Bouillet, Camile Bouisse, Anne-Sophie Boureau, John Bourke, Maude Bouscambert, Aurore Bousquet, Jason Bouziotis, Bianca Boxma, Marielle Boyer-Besseyre, Maria Boylan, Axelle Braconnier, Cynthia Braga, Timo Brandenburger, Filipa Brás Monteiro, Luca Brazzi, Dorothy Breen, Patrick Breen, Kathy Brickell, Alex Browne, Shaunagh Browne, Marjolein Brusse-Keizer, Nina Buchtele, Christian Buesaquillo, Polina Bugaeva, Marielle Buisson, Aidan Burrell, Ingrid G. Bustos, Denis Butnaru, André Cabie, Susana Cabral, Eder Caceres, Cyril Cadoz, Rui Caetano Garcês, Kate Calligy, Jose Andres Calvache, João Camões, Valentine Campana, Paul Campbell, Cecilia Canepa, Mireia Cantero, Pauline Caraux-Paz, Sheila Cárcel, Chiara Simona Cardellino, Filipa Cardoso, Filipe Cardoso, Nelson Cardoso, Sofia Cardoso, Simone Carelli, Nicolas Carlier, Thierry Carmoi, Gayle Carney, Inês Carqueja, Marie-Christine Carret, François Martin Carrier, Ida Carroll, Gail Carson, Maire-Laure Casanova, Mariana Cascão, Siobhan Casey, José Casimiro, Bailey Cassandra, Silvia Castañeda, Nidyanara Castanheira, Guylaine Castor-Alexandre, Henry Castrillón, Ivo Castro, Ana Catarino, François-Xavier Catherine, Paolo Cattaneo, Roberta Cavalin, Giulio Giovanni Cavalli, Alexandros Cavayas, Adrian Ceccato, Minerva Cervantes-Gonzalez, Anissa Chair, Catherine Chakveatze, Adrienne Chan, Meera Chand, Christelle Chantalat Auger, Jean-Marc Chapplain, Julie Chas, Mobin Chaudry, Jonathan Samuel Chávez Iñiguez, Anjellica Chen, Yih-Sharng Chen, Matthew Pellan Cheng, Antoine Cheret, Thibault Chiarabini, Julian Chica, Catherine Chirouze, Davide Chiumello, Sung-Min Cho, Bernard Cholley, Marie-Charlotte Chopin, Jose Pedro Cidade, José Miguel Cisneros Herreros, Anna Ciullo, Emma Clarke, Jennifer Clarke, Sara Clohisey, Perren J. Cobb, Cassidy Codan, Caitriona Cody, Alexandra Coelho, Megan Coles, Gwenhaël Colin, Michael Collins, Sebastiano Maria Colombo, Pamela Combs, Marie Connor, Anne Conrad, Sofía Contreras, Elaine Conway, Graham S. Cooke, Mary Copland, Hugues Cordel, Amanda Corley, Sabine Cornelis, Alexander Daniel Cornet, Arianne Joy Corpuz, Andrea Cortegiani, Grégory Corvaisier, Emma Costigan, Camille Couffignal, Sandrine Couffin-Cadiergues, Roxane Courtois, Stéphanie Cousse, Rachel Cregan, Sabine Croonen, Gloria Crowl, Jonathan Crump, Claudina Cruz, Juan Luis Cruz Bermúdez, Jaime Cruz Rojo, Marc Csete, Ailbhe Cullen, Ger Curley, Elodie Curlier, Colleen Curran, Paula Custodio, Ana da Silva Filipe, Charlene Da Silveira, Al-Awwab Dabaliz, Andrew Dagens, Darren Dahly, Heidi Dalton, Jo Dalton, Seamus Daly, Juliana Damas, Nick Daneman, Corinne Daniel, Emmanuelle A. Dankwa, Jorge Dantas, Frédérick D’Aragon, Gillian de Loughry, Diego de Mendoza, Etienne De Montmollin, Rafael Freitas de Oliveira França, Ana Isabel de Pinho Oliveira, Rosanna De Rosa, Thushan de Silva, Peter de Vries, Jillian Deacon, David Dean, Alexa Debard, Marie-Pierre Debray, Nathalie DeCastro, William Dechert, Lauren Deconninck, Romain Decours, Eve Defous, Isabelle Delacroix, Eric Delaveuve, Karen Delavigne, Andrea Dell’Amore, Pierre Delobel, Corine Delsing, Elisa Demonchy, Emmanuelle Denis, Dominique Deplanque, Pieter Depuydt, Diane Descamps, Mathilde Desvallées, Santi Dewayanti, Pathik Dhanger, Alpha Diallo, Sylvain Diamantis, André Dias, Juan Jose Diaz, Priscila Diaz, Kévin Didier, Jean-Luc Diehl, Wim Dieperink, Jérôme Dimet, Vincent Dinot, Fara Diop, Alphonsine Diouf, Yael Dishon, Félix Djossou, Annemarie B. Docherty, Helen Doherty, Arjen M. Dondorp, Christl A. Donnelly, Maria Donnelly, Chloe Donohue, Sean Donohue, Yoann Donohue, Peter Doran, Céline Dorival, Eric D’Ortenzio, James Joshua Douglas, Nathalie Dournon, Triona Downer, Joanne Downey, Mark Downing, Tom Drake, Aoife Driscoll, Claudio Duarte Fonseca, Vincent Dubee, François Dubos, Alexandre Ducancelle, Susanne Dudman, Paul Dunand, Jake Dunning, Mathilde Duplaix, Emanuele Durante-Mangoni, Lucian Durham, Bertrand Dussol, Juliette Duthoit, Xavier Duval, Anne Margarita Dyrhol-Riise, Marco Echeverria-Villalobos, Siobhan Egan, Carla Eira, Mohammed El Sanharawi, Subbarao Elapavaluru, Brigitte Elharrar, Jacobien Ellerbroek, Philippine Eloy, Tarek Elshazly, Isabelle Enderle, Tomoyuki Endo, Ilka Engelmann, Vincent Enouf, Olivier Epaulard, Martina Escher, Mariano Esperatti, Hélène Esperou, Catarina Espírito Santo, Marina Esposito-Farese, João Estevão, Manuel Etienne, Nadia Ettalhaoui, Anna Greti Everding, Mirjam Evers, Isabelle Fabre, Marc Fabre, Amna Faheem, Arabella Fahy, Cameron J. Fairfield, Zul Fakar, Komal Fareed, Pedro Faria, Ahmed Farooq, Arie Zainul Fatoni, Karine Faure, Raphaël Favory, Mohamed Fayed, Niamh Feely, Laura Feeney, Jorge Fernandes, Marília Andreia Fernandes, Susana Fernandes, François-Xavier Ferrand, Eglantine Ferrand Devouge, Joana Ferrão, Mário Ferraz, Benigno Ferreira, Bernardo Ferreira, Isabel Ferreira, Sílvia Ferreira, Ricard Ferrer-Roca, Nicolas Ferriere, Céline Ficko, Juan Fiorda, Thomas Flament, Clara Flateau, Tom Fletcher, Letizia Lucia Florio, Deirdre Flynn, Claire Foley, Jean Foley, Victor Fomin, Tatiana Fonseca, Patricia Fontela, Simon Forsyth, Denise Foster, Giuseppe Foti, Erwan Fourn, Marianne Fraher, Diego Franch-Llasat, Christophe Fraser, John F. Fraser, Marcela Vieira Freire, Ana Freitas Ribeiro, Craig French, Caren Friedrich, Stéphanie Fry, Nora Fuentes, Masahiro Fukuda, Argin G, Valérie Gaborieau, Rostane Gaci, Massimo Gagliardi, Jean-Charles Gagnard, Amandine Gagneux-Brunon, Sérgio Gaião, Linda Gail Skeie, Phil Gallagher, Carrol Gamble, Arthur Garan, Rebekha Garcia, Noelia García Barrio, Navya Garimella, Denis Garot, Valérie Garrait, Basanta Gauli, Nathalie Gault, Aisling Gavin, Anatoliy Gavrylov, Alexandre Gaymard, Johannes Gebauer, Eva Geraud, Louis Gerbaud Morlaes, Nuno Germano, Jade Ghosn, Marco Giani, Jess Gibson, Tristan Gigante, Morgane Gilg, Elaine Gilroy, Guillermo Giordano, Michelle Girvan, Valérie Gissot, Daniel Glikman, Petr Glybochko, Eric Gnall, Geraldine Goco, François Goehringer, Siri Goepel, Jean-Christophe Goffard, Jonathan Golob, Joan Gómez-Junyent, Marie Gominet, Alicia Gonzalez, Patricia Gordon, Isabelle Gorenne, Laure Goubert, Cécile Goujard, Tiphaine Goulenok, Margarite Grable, Jeronimo Graf, Edward Wilson Grandin, Pascal Granier, Giacomo Grasselli, Christopher A. Green, Courtney Greene, William Greenhalf, Segolène Greffe, Domenico Luca Grieco, Matthew Griffee, Fiona Griffiths, Albert Groenendijk, Anja Grosse Lordemann, Heidi Gruner, Yusing Gu, Jérémie Guedj, Martin Guego, Dewi Guellec, Anne-Marie Guerguerian, Daniela Guerreiro, Romain Guery, Anne Guillaumot, Laurent Guilleminault, Maisa Guimarães de Castro, Thomas Guimard, Marieke Haalboom, Daniel Haber, Hannah Habraken, Ali Hachemi, Nadir Hadri, Fakhir Haidri, Sheeba Hakak, Adam Hall, Matthew Hall, Sophie Halpin, Jawad Hameed, Ansley Hamer, Rebecca Hamidfar, Terese Hammond, Rashan Haniffa, Hayley Hardwick, Ewen M. Harrison, Janet Harrison, Samuel Bernard Ekow Harrison, Mohd Shahnaz Hasan, Junaid Hashmi, Muhammad Hayat, Ailbhe Hayes, Leanne Hays, Jan Heerman, Lars Heggelund, Ross Hendry, Martina Hennessy, Maxime Hentzien, Andrew Hershey, Liv Hesstvedt, Astarini Hidayah, Dawn Higgins, Eibhilin Higgins, Rupert Higgins, Rita Hinchion, Samuel Hinton, Hikombo Hitoto, Antonia Ho, Alexandre Hoctin, Isabelle Hoffmann, Oscar Hoiting, Rebecca Holt, Jan Cato Holter, Peter Horby, Juan Pablo Horcajada, Koji Hoshino, Kota Hoshino, Ikram Houas, Catherine L. Hough, Jimmy Ming-Yang Hsu, Jean-Sébastien Hulot, Stella Huo, Abby Hurd, Iqbal Hussain, Samreen Ijaz, Arfan Ikram, Hajnal-Gabriela Illes, Patrick Imbert, Mohammad Imran, Rana Imran Sikander, Aftab Imtiaz, Hugo Inácio, Carmen Infante Dominguez, Mariachiara Ippolito, Sarah Isgett, Tiago Isidoro, Margaux Isnard, Daniel Ivulich, Danielle Jaafar, Salma Jaafoura, Julien Jabot, Clare Jackson, Nina Jamieson, Pierre Jaquet, Coline Jaud-Fischer, Stéphane Jaureguiberry, Florence Jego, Synne Jenum, Ruth N. Jorge García, Cédric Joseph, Mark Joseph, Swosti Joshi, Mercé Jourdain, Philippe Jouvet, Anna Jung, Dafsah Juzar, Ouifiya Kafif, Florentia Kaguelidou, Sabina Kali, Smaragdi Kalomoiri, Darshana Hewa Kandamby, Chris Kandel, Darakhshan Kanwal, Anant Kataria, Kevin Katz, Christy Kay, Hannah Keane, Seán Keating, Andrea Kelly, Aoife Kelly, Claire Kelly, Niamh Kelly, Sadie Kelly, Yvelynne Kelly, Maeve Kelsey, Kalynn Kennon, Maeve Kernan, Younes Kerroumi, Sharma Keshav, Imrana Khalid, Osama Khalid, Antoine Khalil, Coralie Khan, Irfan Khan, Quratul Ain Khan, Sushil Khanal, Abid Khatak, Amin Khawaja, Michelle E. Kho, Saye Khoo, Nasir Khoso, Yuri Kida, Peter Kiiza, Beathe Kiland Granerud, Anders Benjamin Kildal, Antoine Kimmoun, Detlef Kindgen-Milles, Nobuya Kitamura, Paul Klenerman, Rob Klont, Gry Kloumann Bekken, Stephen R. Knight, Robin Kobbe, Chamira Kodippily, Malte Kohns Vasconcelos, Sabin Koirala, Caroline Kosgei, Arsène Kpangon, Karolina Krawczyk, Oksana Kruglova, Deepali Kumar, Mukesh Kumar, Bharath Kumar Tirupakuzhi Vijayaraghavan, Pavan Kumar Vecham, Dinesh Kuriakose, Ethan Kurtzman, Demetrios Kutsogiannis, Galyna Kutsyna, Konstantinos Kyriakoulis, Marie Lachatre, Marie Lacoste, John G. Laffey, Marie Lagrange, Fabrice Laine, Olivier Lairez, Sanjay Lakhey, Antonio Lalueza, Marc Lambert, François Lamontagne, Marie Langelot-Richard, Vincent Langlois, Eka Yudha Lantang, Marina Lanza, Cédric Laouénan, Samira Laribi, Delphine Lariviere, Stéphane Lasry, Naveed Latif, Odile Launay, Didier Laureillard, Yoan Lavie-Badie, Andrew Law, Cassie Lawrence, Teresa Lawrence, Minh Le, Clément Le Bihan, Cyril Le Bris, Georges Le Falher, Lucie Le Fevre, Quentin Le Hingrat, Marion Le Maréchal, Soizic Le Mestre, Gwenaël Le Moal, Vincent Le Moing, Hervé Le Nagard, Paul Le Turnier, Ema Leal, Marta Leal Santos, James Lee, Jennifer Lee, Su Hwan Lee, Todd C. Lee, Gary Leeming, Bénédicte Lefebvre, Laurent Lefebvre, Benjamin Lefèvre, Sylvie LeGac, Jean-Daniel Lelievre, François Lellouche, Adrien Lemaignen, Véronique Lemee, Anthony Lemeur, Gretchen Lemmink, Jenny Lennon, Rafael León, Marc Leone, Michela Leone, François-Xavier Lescure, Olivier Lesens, Mathieu Lesouhaitier, Amy Lester-Grant, Bruno Levy, Yves Levy, Claire Levy-Marchal, Katarzyna Lewandowska, Erwan L’Her, Gianluigi Li Bassi, Ali Liaquat, Geoffrey Liegeon, Wei Shen Lim, Chantre Lima, Bruno Lina, Andreas Lind, Guillaume Lingas, Sylvie Lion-Daolio, Keibun Liu, Marine Livrozet, Patricia Lizotte, Antonio Loforte, Navy Lolong, Diogo Lopes, Anthony L. Loschner, Paul Loubet, Bouchra Loufti, Guillame Louis, Silvia Lourenco, Lara Lovelace-Macon, Marije Lowik, Jean Christophe Lucet, Carlos Lumbreras Bermejo, Carlos M. Luna, Liem Luong, Nestor Luque, Dominique Luton, Nilar Lwin, Ruth Lyons, Olavi Maasikas, Oryane Mabiala, Moïse Machado, Sara Machado, Gabriel Macheda, Hashmi Madiha, Guillermo Maestro de la Calle, Rafael Mahieu, Sophie Mahy, Ana Raquel Maia, Lars S. Maier, Mylène Maillet, Thomas Maitre, Maximilian Malfertheiner, Nadia Malik, Paddy Mallon, Fernando Maltez, Denis Malvy, Victoria Manda, Laurent Mandelbrot, Julie Mankikian, Edmund Manning, Aldric Manuel, Ceila Maria Sant, Ana Malaque, Daniel Marino, Flávio Marino, Samuel Markowicz, Ana Marques, Catherine Marquis, Brian Marsh, Laura Marsh, Megan Marshal, Celina Turchi Martelli, Emily Martin, Guillaume Martin-Blondel, Martin Martinot, Ana Martins, João Martins, Nuno Martins, Caroline Martins Rego, Gennaro Martucci, Olga Martynenko, Eva Miranda Marwali, David Maslove, Sabina Mason, Sobia Masood, Basri Mat Nor, Basri Mat Nor, Moshe Matan, Meghena Mathew, Daniel Mathieu, Mathieu Mattei, Romans Matulevics, Laurence Maulin, Michael Maxwell, Javier Maynar, Thierry Mazzoni, Natalie Mc Evoy, Lisa Mc Sweeney, Colin McArthur, Colin McArthur, Aine McCarthy, Anne McCarthy, Colin McCloskey, Rachael McConnochie, Sherry McDermott, Sarah E. McDonald, Aine McElroy, Samuel McElwee, Victoria McEneany, Allison McGeer, Chris McKay, Johnny McKeown, Kenneth A. McLean, Paul McNally, Bairbre McNicholas, Elaine McPartlan, Edel Meaney, Cécile Mear-Passard, Maggie Mechlin, Minahel Atif, Maqsood Meher, Ferruccio Mele, Luis Melo, Kashif Memon, Joao Joao Mendes, Ogechukwu Menkiti, Kusum Menon, France Mentré, Alexander J. Mentzer, Emmanuelle Mercier, Noémie Mercier, Antoine Merckx, Mayka Mergeay-Fabre, Blake Mergler, António Mesquita, Osama Metwally, Agnès Meybeck, Dan Meyer, Alison M. Meynert, Vanina Meysonnier, Amina Meziane, Mehdi Mezidi, Céline Michelanglei, Isabelle Michelet, Vladislav Mihnovit, Asma Moin, David Molina, Elena Molinos, Brenda Molloy, Mary Mone, Agostinho Monteiro, Claudia Montes, Giorgia Montrucchio, Sarah Moore, Shona C. Moore, Lina Morales Cely, Lucia Moro, Catherine Motherway, Ana Motos, Hugo Mouquet, Clara Mouton Perrot, Julien Moyet, Aisha Kalsoom Mufti, Jimmy Mullaert, Fredrik Müller, Karl Erik Müller, Daniel Munblit, Syed Muneeb, Nadeem Munir, Aisling Murphy, Aisling Murphy, Lorna Murphy, Marlène Murris, Himed Musaab, Himasha Muvindi, Dimitra Melia Myrodia, Farah Nadia Mohd-Hanafiah, Dave Nagpal, Alex Nagrebetsky, Nageswaran Narayanan, Rashid Nasim Khan, Alasdair Nazerali-Maitland, Nadège Neant, Nikita Nekliudov, Raul Neto, Emily Neumann, Pauline Yeung Ng, Anthony Nghi, Duc Nguyen, Orna Ni Choileain, Niamh Ni Leathlobhair, Prompak Nitayavardhana, Stephanie Nonas, Marion Noret, Lisa Norman, Alessandra Notari, Mahdad Noursadeghi, Adam Nowinski, Saad Nseir, Jose I. Nunez, Nurnaningsih Nurnaningsih, Elsa Nyamankolly, Fionnuala O. Brien, Annmarie O. Callaghan, Annmarie O’Callaghan, Giovanna Occhipinti, Derbrenn OConnor, Max O’Donnell, Tawnya Ogston, Takayuki Ogura, Sophie O’Halloran, Katie O’Hearn, João Oliveira, Larissa Oliveira, David S. Y. Ong, Wilna Oosthuyzen, Anne Opavsky, Peter Openshaw, Saijad Orakzai, Claudia Milena Orozco-Chamorro, Jamel Ortoleva, Javier Osatnik, Linda O’Shea, Miriam O’Sullivan, Nadia Ouamara, Rachida Ouissa, Eric Oziol, Maïder Pagadoy, Justine Pages, Amanda Palacios, Mario Palacios, Massimo Palmarini, Giovanna Panarello, Hem Paneru, Mauro Panigada, Nathalie Pansu, Aurélie Papadopoulos, Rachael Parke, Melissa Parker, Briseida Parra, Taha Pasha, Jérémie Pasquier, Bruno Pastene, Fabian Patauner, Luís Patrão, Patricia Patricio, Juliette Patrier, Lisa Patterson, Rajyabardhan Pattnaik, Christelle Paul, Mical Paul, Jorge Paulos, William A. Paxton, Jean-François Payen, Florent Peelman, Nathan Peiffer-Smadja, Vincent Peigne, Mare Pejkovska, Paolo Pelosi, Ithan D. Peltan, Rui Pereira, Daniel Perez, Luis Periel, Thomas Perpoint, Antonio Pesenti, Vincent Pestre, Lenka Petrou, Michele Petrovic, Ventzislava Petrov-Sanchez, Frank Olav Pettersen, Gilles Peytavin, Scott Pharand, Walter Picard, Olivier Picone, Carola Pierobon, Djura Piersma, Carlos Pimentel, Raquel Pinto, Catarina Pires, Isabelle Pironneau, Lionel Piroth, Riinu Pius, Laurent Plantier, Julien Poissy, Ryadh Pokeerbux, Sergio Poli, Georgios Pollakis, Diane Ponscarme, Andra-Maris Post, Douwe F. Postma, Pedro Povoa, Diana Póvoas, Jeff Powis, Sofia Prapa, Sébastien Preau, Christian Prebensen, Jean-Charles Preiser, Anton Prinssen, Mark G. Pritchard, Gamage Dona Dilanthi Priyadarshani, Lucia Proença, Sravya Pudota, Oriane Puéchal, Bambang Pujo Semedi, Mathew Pulicken, Gregory Purcell, Luisa Quesada, Vilmaris Quinones-Cardona, Víctor Quirós González, Else Quist-Paulsen, Mohammed Quraishi, Christian Rabaud, Ebenezer Rabindrarajan, Aldo Rafael, Marie Rafiq, Arsalan Rahutullah, Fernando Rainieri, Pratheema Ramachandran, Nagarajan Ramakrishnan, José Ramalho, Blandine Rammaert, Grazielle Viana Ramos, Asim Rana, Rajavardhan Rangappa, Ritika Ranjan, Christophe Rapp, Aasiyah Rashan, Thalha Rashan, Ghulam Rasheed, Menaldi Rasmin, Indrek Rätsep, Cornelius Rau, Ali Raza, Andre Real, Stanislas Rebaudet, Sarah Redl, Brenda Reeve, Attaur Rehman, Liadain Reid, Liadain Reid, Dag Henrik Reikvam, Renato Reis, Jonathan Remppis, Martine Remy, Hongru Ren, Hanna Renk, Anne-Sophie Resseguier, Matthieu Revest, Oleksa Rewa, Tiago Reyes, Maria Ines Ribeiro, David Richardson, Denise Richardson, Laurent Richier, Jordi Riera, Ana L. Rios, Asgar Rishu, Patrick Rispal, Karine Risso, Nicholas Rizer, Chiara Robba, André Roberto, Stephanie Roberts, David L. Robertson, Olivier Robineau, Ferran Roche-Campo, Paola Rodari, Simão Rodeia, Julia Rodriguez Abreu, Bernhard Roessler, Pierre-Marie Roger, Amanda Rojek, Juliette Romaru, Roberto Roncon-Albuquerque, Mélanie Roriz, Manuel Rosa-Calatrava, Michael Rose, Dorothea Rosenberger, Andrea Rossanese, Matteo Rossetti, Bénédicte Rossignol, Patrick Rossignol, Stella Rousset, Carine Roy, Benoît Roze, Desy Rusmawatiningtyas, Clark D. Russell, Maeve Ryan, Maria Ryan, Steffi Ryckaert, Aleksander Rygh Holten, Isabela Saba, Sairah Sadaf, Musharaf Sadat, Valla Sahraei, Nadia Saidani, Maximilien Saint-Gilles, Pranya Sakiyalak, Nawal Salahuddin, Leonardo Salazar, Jodat Saleem, Gabriele Sales, Stéphane Sallaberry, Charlotte Salmon Gandonniere, Hélène Salvator, Olivier Sanchez, Angel Sanchez-Miralles, Vanessa Sancho-Shimizu, Gyan Sandhu, Zulfiqar Sandhu, Pierre-François Sandrine, Oana Sandulescu, Marlene Santos, Shirley Sarfo-Mensah, Bruno Sarmento Banheiro, Benjamine Sarton, Sree Satyapriya, Rumaisah Satyawati, Egle Saviciute, Justin Schaffer, Tjard Schermer, Arnaud Scherpereel, Marion Schneider, Stephan Schroll, Michael Schwameis, Janet T. Scott, James Scott-Brown, Nicholas Sedillot, Tamara Seitz, Jaganathan Selvanayagam, Caroline Semaille, Malcolm G. Semple, Eric Senneville, Filipa Sequeira, Tânia Sequeira, Ary Serpa Neto, Pablo Serrano Balazote, Ellen Shadowitz, Mohammad Shamsah, Shaikh Sharjeel, Pratima Sharma, Catherine A. Shaw, Victoria Shaw, Ashraf Sheharyar, Rajesh Mohan Shetty, Haixia Shi, Mohiuddin Shiekh, Keiki Shimizu, Sally Shrapnel, Shubha Kalyan Shrestha, Pramesh Sundar Shrestha, Hoi Ping Shum, Nassima Si Mohammed, Jeanne Sibiude, Atif Siddiqui, Louise Sigfrid, Piret Sillaots, Catarina Silva, Maria Joao Silva, Rogério Silva, Wai Ching Sin, Budha Charan Singh, Punam Singh, Pompini Agustina Sitompul, Vegard Skogen, Sue Smith, Benjamin Smood, Coilin Smyth, Michelle Smyth, Michelle Smyth, Morgane Snacken, Dominic So, Joshua Solomon, Tom Solomon, Emily Somers, Agnès Sommet, Myung Jin Song, Rima Song, Tae Song, Jack Song Chia, Albert Sotto, Edouard Soum, Ana Chora Sousa, Marta Sousa, Maria Sousa Uva, Alexandra Sperry, Elisabetta Spinuzza, B. P. Sanka Ruwan Sri Darshana, Shiranee Sriskandan, Sarah Stabler, Thomas Staudinger, Stephanie-Susanne Stecher, Ymkje Stienstra, Birgitte Stiksrud, Eva Stolz, Amy Stone, Adrian Streinu-Cercel, Anca Streinu-Cercel, Ami Stuart, David Stuart, Gabriel Suen, Jacky Y. Suen, Asfia Sultana, Charlotte Summers, Dubravka Supic, Magdalena Surovcová, Andrey Svistunov, Konstantinos Syrigos, Jaques Sztajnbok, Konstanty Szuldrzynski, Shirin Tabrizi, Lysa Tagherset, Sara Taleb, Jelmer Talsma, Maria Lawrensia Tampubolon, Hiroyuki Tanaka, Huda Taqdees, Arshad Taqi, Coralie Tardivon, Pierre Tattevin, M. Azhari Taufik, Hassan Tawfik, Richard S. Tedder, João Teixeira, Sofia Tejada, Marie-Capucine Tellier, François Téoulé, Pleun Terpstra, Olivier Terrier, Nicolas Terzi, Hubert Tessier-Grenier, Adrian Tey, Anand Thakur, Vincent Thibault, Simon-Djamel Thiberville, Benoît Thill, Shaun Thompson, Emma C. Thomson, Ryan S. Thwaites, Paul Tierney, Vadim Tieroshyn, Peter S. Timashev, Jean-François Timsit, Bharath Kumar Tirupakuzhi Vijayaraghavan, Noémie Tissot, Maria Toki, Kristian Tonby, Marta Torre, Antoni Torres, Margarida Torres, Hernando Torres-Zevallos, Michael Towers, Tony Trapani, Théo Treoux, Cécile Tromeur, Ioannis Trontzas, Tiffany Trouillon, Jeanne Truong, Christelle Tual, Sarah Tubiana, Helen Tuite, Jean-Marie Turmel, Lance C. W. Turtle, Pawel Twardowski, Makoto Uchiyama, P. G. Ishara Udayanga, Andrew Udy, Roman Ullrich, Alberto Uribe, Asad Usman, Timothy M. Uyeki, Cristinava Vajdovics, Luís Val-Flores, Amélie Valran, Stijn Van de Velde, Marcel van den Berge, Job van der Palen, Paul van der Valk, Nicky Van Der Vekens, Peter Van der Voort, Sylvie Van Der Werf, Laura van Gulik, Jarne Van Hattem, Carolien van Netten, Ilonka van Veen, Noémie Vanel, Henk Vanoverschelde, Pooja Varghese, Charline Vauchy, Aurélie Veislinger, Sebastian Vencken, Sara Ventura, Annelies Verbon, James Vickers, José Ernesto Vidal, César Vieira, Deepak Vijayan, Joy Ann Villanueva, Judit Villar, Pierre-Marc Villeneuve, Andrea Villoldo, Benoit Visseaux, Hannah Visser, Chiara Vitiello, Harald Vonkeman, Fanny Vuotto, Wan Fadzlina Wan Muhd Shukeri, Chih-Hsien Wang, Steve Webb, Jia Wei, Katharina Weil, Sanne Wesselius, T. Eoin West, Murray Wham, Bryan Whelan, Nicole White, Paul Henri Wicky, Aurélie Wiedemann, Surya Otto Wijaya, Keith Wille, Suzette Willems, Virginie Williams, Evert-Jan Wils, Ng Wing Yiu, Calvin Wong, Ioannis Xynogalas, Masaki Yamazaki, Yazdan Yazdanpanah, Cécile Yelnik, Stephanie Yerkovich, Toshiki Yokoyama, Hodane Yonis, Obada Yousif, Saptadi Yuliarto, Akram Zaaqoq, Marion Zabbe, Maram Zahran, Maria Zambon, Alberto Zanella, Hiba Zayyad, Alexander Zoufaly, David Zucman

**Affiliations:** 1 Pandemic Sciences Institute, University of Oxford, Oxford, UK; 2 Infectious Diseases Department, Universidad de La Sabana, Chía, Colombia; 3 Critical Care Department, Clínica Universidad de La Sabana, Chía, Colombia; 4 Department of Pediatrics, University of British Columbia, Vancouver, Canada; 5 Clinical Research/Epidemiology in Pneumonia & Sepsis (CRIPS), Vall d’Hebron Institute of Research (VHIR), Barcelona, Spain; 6 Centro de Investigación Biomédica En Red de Enfermedades Respiratorias (CIBERES), Instituto de Salud Carlos III, Madrid, Spain; 7 Department of Clinical Medicine, St James’s Hospital, Multidisciplinary Intensive Care Research Organization (MICRO), Dublin, Ireland; 8 D’Or Institute for Research and Education (IDOR), Rio de Janeiro, RJ Brazil; 9 Brazilian Research in Intensive Care Network (BRICNet), Rio de Janeiro, Brazil; 10 Oswaldo Cruz Foundation (FIOCRUZ), Rio de Janeiro, RJ Brazil; 11 Department of Intensive Care, Université Libre de Bruxelles (ULB), Brussels, Belgium; 12 Laboratoire de Recherche Experimentale, Department of Intensive Care, Hôpital Erasme, Brussels, Belgium; 13 Interdepartmental Division of Critical Care Medicine, University of Toronto, Toronto, ON Canada; 14 Nepal Mediciti Hospital, Lalitpur, Nepal; 15 Infection Division, Department of Pulmonology and Respiratory Medicine, Universitas Indonesia, Depok, Indonesia; 16 Division of Pulmonary, Allergy, and Critical Care Medicine, Department of Medicine, Columbia University Vagelos College of Physicians and Surgeons, New York, NY USA; 17 IAME, INSERM, Paris, France; 18 Intensive Care Unit, Clinica Las Condes, Santiago, Chile; 19 Instituto de Infectologia Emílio Ribas, São Paulo, Brazil; 20 Critical Care Asia and Ziauddin University, Karachi, Pakistan; 21 All India Institute of Medical Sciences (AIIMS), Rishikesh, India; 22 Hospital 12 de Octubre, Madrid, Spain; 23 Department of Intensive Care, Fundación Valle del Lili, Cali, Colombia; 24 Division of Critical Care, University of Cape Town and Groote Schuur Hospital, Cape Town, South Africa; 25 University College Dublin Clinical Research Centre at St Vincent’s University Hospital, Dublin, Ireland; 26 Li Ka Shing Knowledge Institute, Unity Health Toronto, St Michael’s Hospital, Toronto, ON Canada

**Keywords:** Invasive mechanical ventilation, High flow nasal cannula, COVID-19, Critical care

## Abstract

**Background:**

Up to 30% of hospitalised patients with COVID-19 require advanced respiratory support, including high-flow nasal cannulas (HFNC), non-invasive mechanical ventilation (NIV), or invasive mechanical ventilation (IMV). We aimed to describe the clinical characteristics, outcomes and risk factors for failing non-invasive respiratory support in patients treated with severe COVID-19 during the first two years of the pandemic in high-income countries (HICs) and low middle-income countries (LMICs).

**Methods:**

This is a multinational, multicentre, prospective cohort study embedded in the ISARIC-WHO COVID-19 Clinical Characterisation Protocol. Patients with laboratory-confirmed SARS-CoV-2 infection who required hospital admission were recruited prospectively. Patients treated with HFNC, NIV, or IMV within the first 24 h of hospital admission were included in this study. Descriptive statistics, random forest, and logistic regression analyses were used to describe clinical characteristics and compare clinical outcomes among patients treated with the different types of advanced respiratory support.

**Results:**

A total of 66,565 patients were included in this study. Overall, 82.6% of patients were treated in HIC, and 40.6% were admitted to the hospital during the first pandemic wave. During the first 24 h after hospital admission, patients in HICs were more frequently treated with HFNC (48.0%), followed by NIV (38.6%) and IMV (13.4%). In contrast, patients admitted in lower- and middle-income countries (LMICs) were less frequently treated with HFNC (16.1%) and the majority received IMV (59.1%). The failure rate of non-invasive respiratory support (i.e. HFNC or NIV) was 15.5%, of which 71.2% were from HIC and 28.8% from LMIC. The variables most strongly associated with non-invasive ventilation failure, defined as progression to IMV, were high leukocyte counts at hospital admission (OR [95%CI]; 5.86 [4.83–7.10]), treatment in an LMIC (OR [95%CI]; 2.04 [1.97–2.11]), and tachypnoea at hospital admission (OR [95%CI]; 1.16 [1.14–1.18]). Patients who failed HFNC/NIV had a higher 28-day fatality ratio (OR [95%CI]; 1.27 [1.25–1.30]).

**Conclusions:**

In the present international cohort, the most frequently used advanced respiratory support was the HFNC. However, IMV was used more often in LMIC. Higher leucocyte count, tachypnoea, and treatment in LMIC were risk factors for HFNC/NIV failure. HFNC/NIV failure was related to worse clinical outcomes, such as 28-day mortality.

*Trial registration* This is a prospective observational study; therefore, no health care interventions were applied to participants, and trial registration is not applicable.

**Supplementary Information:**

The online version contains supplementary material available at 10.1186/s13054-022-04155-1.

## Background

The Severe Acute Respiratory Syndrome Coronavirus 2 (SARS-CoV-2) has infected over 500 million people worldwide and resulted in more than 6 million deaths (https://covid19.who.int) [1, 2]. COVID-19, the disease caused by the SARS-CoV-2, is a multisystemic disease [3]. Its most severe presentation is acute respiratory distress syndrome (ARDS), secondary to pneumonia [4–6]. Most critically ill patients with COVID-19 receive advanced respiratory support, defined as high-flow nasal cannula (HFNC), non-invasive mechanical ventilation (NIV), or invasive mechanical ventilation (IMV) [3, 7, 8]. Up to 30% of hospitalised patients with COVID-19 are treated with one of these interventions [9, 10]; however, the use and need for support have changed over time depending on COVID-19 vaccination coverage, circulating viral variants, an evolving treatment evidence base and practice variation [11, 12].

Given the high demand for respiratory support and the insufficient capacity of intensive care units (ICU) and resources during the pandemic, especially in low- and middle-income countries (LMIC), the use of less invasive alternatives emerged as an alternative to provide advanced respiratory support [13, 14]. A global survey in 2020 found that HFNC (54%) and NIV (47%) were the most frequently used types of advanced respiratory support in patients with severe COVID-19 [15]. Up to 37% of patients who received NIV support ultimately required IMV [16], with high fatality ratios, especially in Latin America [17].

The objectives of this global study are to describe the clinical characteristics and outcomes of patients treated with HFNC, NIV, and IMV during the first two years of the pandemic, to determine risk factors associated with HFNC and NIV failure, and to estimate the association of later administration of IMV on clinical outcomes. We also compare the respiratory support types used in high-income countries (HICs) with those used in LMIC.

## Methods

This is a prospective observational study of hospitalised patients from five continents. The study Consortium framework is provided by the International Severe Acute Respiratory and Emerging Infection (ISARIC)—World Health Organization (WHO) Clinical Characterisation Protocol for Severe Emerging Infections [18, 19]. The protocol, case report forms, consent forms, and study information are available on the ISARIC website (https://isaric.org). This standardised protocol uses tiered data collection tailored to a range of resource settings [19]. Investigators from 69 countries collected prospective data using the ISARIC case report form (CRF) built on Research Electronic Data Capture (REDCap, version 8.11.11, Vanderbilt University, Nashville, Tenn.) [20] hosted by the University of Oxford. Other investigators collected data using locally hosted systems and submitted it to the ISARIC dataset for centralised mapping. All investigators retain full rights to their data.

This observational study required no change to clinical management and encouraged patient enrolment in other research projects. The ISARIC-WHO Clinical Characterisation Protocol was approved by the World Health Organization Ethics Review Committee (RPC571 and RPC572). Also, local ethics approval was obtained for each participating country and site according to local requirements.

### Study population

We included hospitalised patients with confirmed SARS-CoV-2 infection by reverse transcription-polymerase chain reaction (RT-PCR) in a respiratory sample treated with advanced respiratory support, defined as either HFNC, NIV, or IMV [3]. Patients with no recorded demographic data or vital signs within the first 24 h of hospital admission were excluded, as were patients whose 28-day vital status was unknown.

### Variables and measurement

We recorded age, sex, income classification according to the World Bank (https://data.worldbank.org/country) of the country of recruitment, comorbidities, vital signs on admission, laboratory measurements during the first 24 h of hospital admission, treatment with advanced respiratory support at any point during hospitalisation, systemic complications, and treatments used during hospitalisation. The case report form completion guide is available online (https://isaric.org).

We identified the first wave of the pandemic for each participating country and composed a dichotomous variable to evaluate the impact of being admitted during the first wave on clinical outcomes.

We stratified patients in the cohort based on the first type of respiratory support received within the first 24 h of hospital admission. *High-flow nasal cannula (HFNC)* was defined as respiratory support continuously applied through large-bore nasal prongs using a heated and humid gas flow at an initial flow more significant than 20 L/min (or up to 80 L per minute) and a fraction of inspired oxygen of up to 1.0. *Non-invasive mechanical ventilation (NIV)* was defined as any type of positive pressure therapy delivered through a fitted mask and was preferred in patients with oxygen requirements over 6–15 L/min or laboured breathing. Continuous positive pressure (CPAP) or bi-positive pressure (BiPAP) may occur and be considered NIV. *Invasive mechanical ventilation (IMV)* is any mechanical ventilation administered to the patient after endotracheal intubation or tracheostomy. The decision to use this modality was left to the health care providers and not per study protocol.

Patients were considered to have failed the non-invasive respiratory strategy (i.e. HFNC or NIV) if they were subsequently treated with IMV during hospitalisation.

### Outcomes

The primary outcome evaluated in this study was 28-day mortality. Secondary outcomes included the rate of and risk factors for failing non-invasive respiratory support (i.e. HFNC or NIV), the association of failure with clinical outcomes, and the frequency of respiratory strategies used in HIC versus LMICs.

### Statistical methods

Continuous variables were expressed as median (interquartile range), and categorical variables as counts (percentages). For the primary outcome of 28-day mortality and secondary outcome of non-invasive respiratory failure, random forest (RF) models were used to identify the factors associated with these outcomes. The RF model uses multiple randomised individual decision trees that operate as an ensemble, where each decision tree gives a predicted class. The class obtained most frequently among the decision trees becomes the RF model prediction. A total of 500 estimators were used in this model. A more detailed explanation of the RF models is presented in the supplement.

To evaluate the performance of the RF model, the area under the model's receiver operating characteristics curve (AUROC) was used; for this, a tenfold cross-validation method was used, in which the data set was divided into ten subsets, and the validation was repeated ten times. Each time, one of the subsets was used as the test cohort, and the other nine subsets were used as training subsets, then the average AUROC was calculated and reported. When used for classification, RF models perform an implicit feature selection, a general indicator of each specific feature relevance, and can be computed as the Gini importance.

Then, we fitted two multivariable logistic regression models to estimate associations with the risk of 28-day fatality ratio or non-invasive respiratory failure, respectively. Variables identified as relevant by the RF model were included as explanatory variables. Odds ratios (ORs) were presented with forest plots.

A patient treated with respiratory support might receive different strategies during hospital admission. Thus, we developed alluvia diagrams to understand how patients were treated with other respiratory methods over time, stratified by the countries’ income classification. We constructed chord diagrams to provide a graphical representation of these patients' comorbid conditions and demographics differentiated by the income classification. A significance level of < 0.001 and a confidence level of 95% was chosen to determine statistical differences. This was selected as large datasets, such as the ISARIC COVID-19 dataset, might identify minor differences as significant even when the differences are not clinically relevant. Adjusting the rejection level of the null hypothesis could control this limitation inherent to large datasets and the possibility of incurring type one error. All data processing and statistical analysis were performed using Python version 3.8 with the following data packages: Pandas version 1.2.4, Tidyverse version 1.3.0, Bioconductor version 3.12.

## Results

**Fig. 1:**
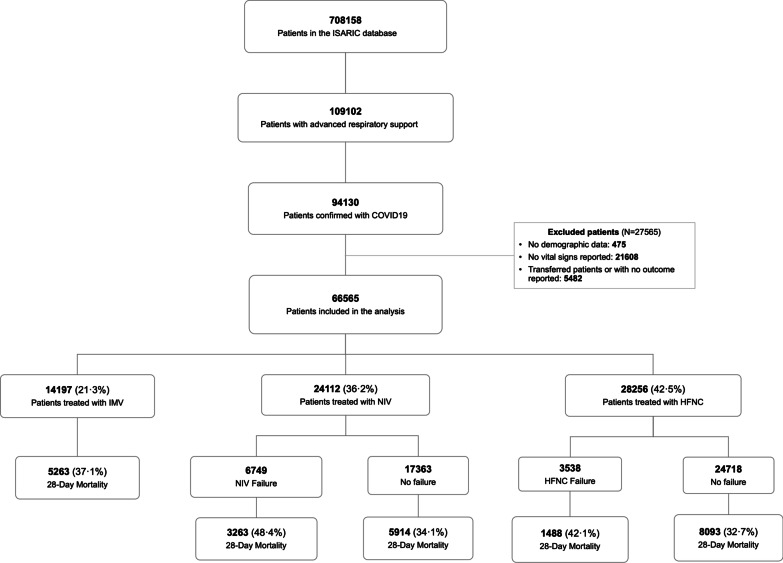
Flow chart. This figure shows patients included in the analysis and cohort selection process

**Fig. 2:**
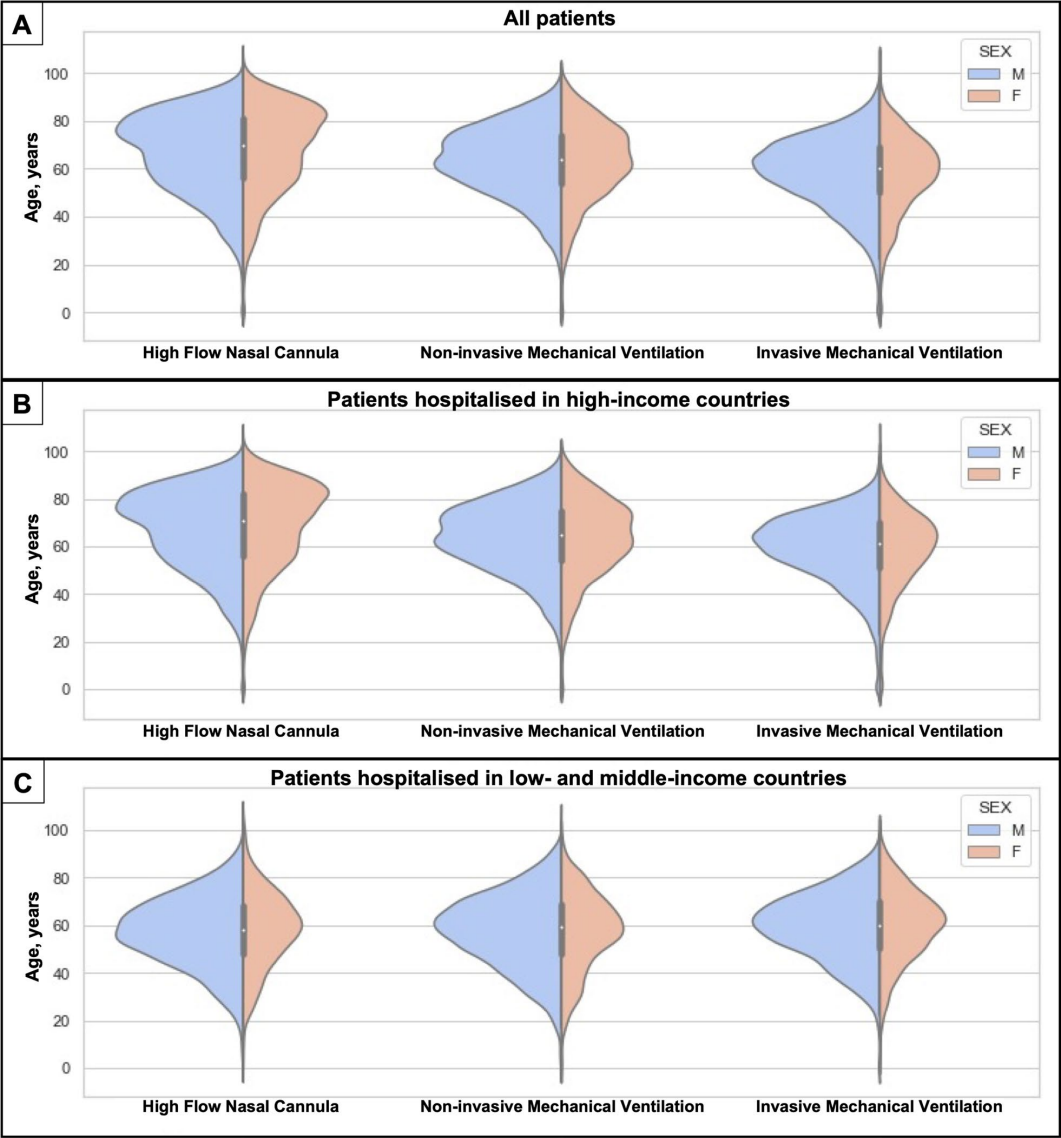
Probability density of patients' basic demographics (age and sex), according to the first ventilation treatment received. **A** Complete cohort. **B** Patients from high-income countries. **C** Patients from low middle-income countries

A total of 66,565 patients were included in this study (Fig. 1). Most patients were male (63.5% [42,256/66,565]) and treated in HICs (82.6% [55,004/66,565]). Specifically, 78.2% ([52,039/66,565]) of the cohort was hospitalised and treated in Europe. Regarding the age of the patients included in the cohort, 44.0% ([29,317/66,565]) of patients were between 60 and 80 years old. During the first 24 h of hospital admission, patients were most frequently treated with HFNC (42.5% [28,256/66,565]), followed by NIV (36.2% [24,112/66,565]) and IMV (21.3% [14,197/66,565]). Demographic characteristics, physiological variables and laboratories at hospital admission are shown in Fig. 2 and Tables 1 and 2.

**Table 1:** Baseline characteristics of patients, stratified by the different advance ventilatory supports

Characteristic	All *n* = 66,565	HFNC *n* = 28,256	NIV *n* = 24,112	IMV *n* = 14,197	*p* value
*Demographics, n (%)*					
Female	24,309 (36.5)	11,188 (39.6)	8600 (35.7)	4521 (31.8)	** < 0.001**
Age 0–20 years old	558 (0.9)	188 (0.7)	156 (0.6)	214 (1.5)	** < 0.001**
Age 20–40 years old	4888 (7.4)	1800 (6.3)	1635 (6.8)	1453 (10.2)	** < 0.001**
Age 40–60 years old	19,514 (29.3)	6907 (24.5)	7429 (30.8)	5178 (36.5)	** < 0.001**
Age 60–80 years old	29,317 (44.0)	11,350 (40.2)	11,505 (47.7)	6462 (45.5)	** < 0.001**
Age 80–100 years old	12,232 (18.4)	7962 (28.1)	3382 (14.1)	888 (6.2)	** < 0.001**
Age ≥ 100 years old	56 (0.1)	49 (0.2)	5 (0.0)	2 (0.0)	** < 0.001**
*Pandemic wave in which patients were admitted, n (%)*					
First COVID-19 wave	27,044 (40.6)	13,363 (47.3)	7888 (32.7)	5793 (40.8)	** < 0.001**
*Continent of admission, n (%)*					
Africa	89 (0.1)	17 (0.1)	3 (0.0)	69 (0.5)	** < 0.001**
Asia	10,488 (15.8)	1520 (5.4)	2590 (10.7)	6378 (44.9)	** < 0.001**
Europe	52,039 (78.2)	25,586 (90.6)	20,924 (86.8)	5529 (38.9)	** < 0.001**
North America	2434 (3.7)	561 (2.0)	277 (1.1)	1596 (11.2)	** < 0.001**
Oceania	260 (0.4)	155 (0.5)	8 (0.0)	97 (0.7)	** < 0.001**
South America	1255 (1.9)	417 (1.5)	310 (1.3)	528 (3.7)	** < 0.001**
*Regional income stratification, n (%)*					
High-income country	55,004 (82.6)	26,399 (93.4)	21,237 (88.1)	7368 (51.9)	** < 0.001**
Low middle-income country	11,561 (17.4)	1857 (6.6)	2875 (11.9)	6829 (48.1)	** < 0.001**
*Chronic comorbidities, n (%)*					
Asthma	8097 (12.2)	3596 (12.7)	3413 (14.2)	1088 (7.7)	** < 0.001**
Chronic cardiac disease (not hypertension)	14,678 (22.1)	7794 (27.6)	5153 (21.4)	1731 (12.2)	** < 0.001**
Chronic kidney disease	7533 (11.3)	4135 (14.6)	2571 (10.7)	827 (5.8)	** < 0.001**
Chronic neurological disorder	4944 (7.4)	2808 (9.9)	1560 (6.5)	576 (4.1)	** < 0.001**
Chronic pulmonary disease (not asthma)	8856 (13.3)	4459 (15.8)	3551 (14.7)	846 (6.0)	** < 0.001**
Dementia	3964 (6.0)	3032 (10.7)	818 (3.4)	114 (0.8)	** < 0.001**
Diabetes mellitus	20,164 (30.3)	8273 (29.3)	7343 (30.5)	4548 (32.0)	** < 0.001**
HIV	271 (0.4)	105 (0.4)	93 (0.4)	73 (0.5)	0.08
Arterial hypertension	27,521 (41.3)	11,855 (42.0)	9874 (41.0)	5792 (40.8)	0.02
Hypothyroidism	1632 (2.5)	864 (3.1)	598 (2.5)	170 (1.2)	** < 0.001**
Immunosuppression	1242 (1.9)	659 (2.3)	491 (2.0)	92 (0.6)	** < 0.001**
Malignant neoplasm	5115 (7.7)	2811 (9.9)	1803 (7.5)	501 (3.5)	** < 0.001**
Malnutrition	894 (1.3)	565 (2.0)	229 (0.9)	100 (0.7)	** < 0.001**
Mental disorder	1042 (1.6)	541 (1.9)	418 (1.7)	83 (0.6)	** < 0.001**
Moderate or severe liver disease	880 (1.3)	465 (1.6)	282 (1.2)	133 (0.9)	** < 0.001**
Obesity	10,793 (16.2)	3960 (14.0)	4883 (20.3)	1950 (13.7)	** < 0.001**
Rheumatological disorder	5412 (8.1)	3033 (10.7)	1989 (8.2)	390 (2.7)	** < 0.001**
Smoking	15,190 (22.8)	6948 (24.6)	6521 (27.0)	1721 (12.1)	** < 0.001**
Solid tumour	522 (0.8)	307 (1.1)	186 (0.8)	29 (0.2)	** < 0.001**
*Complications, n (%)*					
Acute kidney injury	13,353 (20.1)	5146 (18.2)	4525 (18.8)	3682 (25.9)	** < 0.001**
Anaemia	10,031 (15.1)	3803 (13.5)	3492 (14.5)	2736 (19.3)	** < 0.001**
ARDS	15,470 (23.2)	4846 (17.2)	5625 (23.3)	4999 (35.2)	** < 0.001**
Bacteraemia	3966 (6.0)	1191 (4.2)	1381 (5.7)	1394 (9.8)	** < 0.001**
Cardiac arrest	3882 (5.8)	1215 (4.3)	1275 (5.3)	1392 (9.8)	** < 0.001**
Cardiac arrhythmia	5989 (9.0)	2070 (7.3)	2208 (9.2)	1711 (12.1)	** < 0.001**
Cardiac ischemia	1175 (1.8)	471 (1.7)	426 (1.8)	278 (2.0)	0.10
Coagulation disorder	3231 (4.9)	1122 (4.0)	1346 (5.6)	763 (5.4)	** < 0.001**
Congestive heart failure	2188 (3.3)	1159 (4.1)	749 (3.1)	280 (2.0)	** < 0.001**
Gastrointestinal bleeding	1130 (1.7)	519 (1.8)	310 (1.3)	301 (2.1)	** < 0.001**
Liver dysfunction	5600 (8.4)	1972 (7.0)	2176 (9.0)	1452 (10.2)	** < 0.001**
Neurological complication	1206 (1.8)	522 (1.8)	458 (1.9)	226 (1.6)	0.08
Pleural effusion	3967 (6.0)	1858 (6.6)	1285 (5.3)	824 (5.8)	** < 0.001**
Pneumothorax	1590 (2.4)	458 (1.6)	671 (2.8)	461 (3.2)	** < 0.001**
Pulmonary embolism	1951 (2.9)	667 (2.4)	869 (3.6)	415 (2.9)	** < 0.001**
Stroke	918 (1.4)	358 (1.3)	302 (1.3)	258 (1.8)	** < 0.001**
*Treatments, n (%)*					
Prone	15,778 (23.7)	3384 (12.0)	6628 (27.5)	5766 (40.6)	** < 0.001**
Vasopressors/inotropes	13,592 (20.4)	2188 (7.7)	4282 (17.8)	7122 (50.2)	** < 0.001**
Corticoids	40,810 (61.3)	15,586 (55.2)	17,043 (70.7)	8181 (57.6)	** < 0.001**
Intensive care unit	36,336 (54.6)	8302 (29.4)	14,180 (58.8)	13,854 (97.6)	** < 0.001**
*Clinical outcomes*					
Hospital discharge	33,627 (50.5)	16,302 (57.7)	12,115 (50.2)	5210 (36.7)	** < 0.001**
28-day fatality ratio	24,021 (36.1)	9581 (33.9)	9177 (38.1)	5263 (37.1)	** < 0.001**
Non-invasive ventilation failure (HFNC and NIV)	10,287 (15.5)	3538 (12.5)	6749 (28.0)		** < 0.001**

Bold values indicate statistical significance

*HFNC* high-flow nasal cannula, *NIV* non-invasive mechanical ventilation, *IMV* invasive mechanical ventilation, *HIV* human immunodeficiency virus, *ARDS* acute respiratory distress syndrome

**Table 2:** Physiological parameters and laboratories of patients during the first 24-h hospital admission, stratified by the different advance ventilatory supports

Measure	All		HFNC		NIV		IMV		*p* value
	Value	*n*	Value	*n*	Value	*n*	Value	*n*	
*Physiological parameters on admission, median (IQR)*									
Diastolic blood pressure (mmHg)	74.0 (65.0–83.0)	64,667	74.0 (65.0–83.0)	27,806	75.0 (66.0–83.0)	23,713	73.0 (64.0–82.0)	13,148	** < 0.001**
Heart rate (beats/min)	92.0 (80.0–106.0)	64,563	91.0 (80.0–105.0)	27,564	94.0 (82.0–107.0)	23,756	93.0 (81.0–108.0)	13,243	** < 0.001**
Respiratory rate (breaths/min)	24.0 (20.0–28.0)	63,891	22.0 (20.0–28.0)	27,631	24.0 (20.0–30.0)	23,439	24.0 (20.0–28.0)	12,821	** < 0.001**
Systolic blood pressure (mmHg)	129.0 (115.0–144.0)	64,745	129.0 (114.0–144.0)	27,828	130.0 (116.0–145.0)	23,740	129.0 (113.0–142.0)	13,177	** < 0.001**
Temperature (C)	37.2 (36.7–38.1)	64,519	37.2 (36.6–38.1)	27,763	37.3 (36.7–38.2)	23,685	37.0 (36.7–37.8)	13,071	** < 0.001**
*Laboratory during the first 24 h, median (IQR)*									
Alanine aminotransferase (U/L)	32.0 (21.0–53.0)	28,901	29.0 (19.0–48.0)	13,612	34.0 (22.0–55.0)	10,956	38.0 (25.0–62.6)	4333	** < 0.001**
Aspartate aminotransferase (U/L)	50.0 (33.0–77.0)	6614	47.0 (31.0–73.0)	2413	51.0 (34.0–78.0)	2085	51.0 (34.0–81.0)	2116	** < 0.001**
Base excess (mmol/L)	0.0 (− 2.8–2.5)	7017	0.5 (− 2.1–2.9)	2542	0.3 (− 2.2–2.6)	2379	− 1.0 (− 4.0–1.9)	2096	** < 0.001**
Bicarbonate (mEq/L)	23.0 (20.4–25.8)	9601	23.5 (20.9–25.9)	2254	23.1 (20.5–26.0)	2536	23.0 (20.0–25.7)	4811	** < 0.001**
Bilirubin (umol/L)	10.0 (7.0–14.0)	29,034	9.0 (7.0–14.0)	13,705	10.0 (7.0–14.0)	11,029	9.0 (6.0–15.0)	4300	** < 0.001**
C reactive protein (mg/L)	106.0 (53.0–179.0)	34,518	94.4 (48.0–161.55)	16,762	118.0 (63.0–191.0)	13,899	118.5 (45.63–213.7)	3857	** < 0.001**
Creatine kinase (U/L)	163.0 (76.0–427.75)	4702	151.5 (67.75–409.0)	1880	164.0 (80.0–431.0)	1557	179.0 (83.0–450.0)	1265	0.001
Creatinine (umol/L)	87.0 (68.95–120.0)	42,151	87.0 (69.0–120.0)	17,420	87.0 (69.0–116.0)	14,859	87.52 (67.19–126.41)	9872	0.20
D-Dimer (mg/L)	2.76 (0.9–375.0)	1395	1.54 (0.75–9.82)	357	6.94 (0.89–607.0)	303	3.68 (1.02–453.0)	735	** < 0.001**
Ferritin (ug/L)	810.0 (384.0–1523.0)	6244	735.0 (326.0–1425.7)	2542	838.0 (411.92–1535.5)	2460	942.0 (447.0–1672.75)	1242	** < 0.001**
Glucose (mmol/L)	7.55 (6.2–10.4)	33,299	7.0 (5.9–9.3)	12,720	7.6 (6.3–10.5)	12,543	8.49 (6.83–11.84)	8036	** < 0.001**
Haematocrit (%)	39.0 (34.6–42.3)	11,810	39.7 (35.1–43.0)	2389	39.0 (35.0–42.8)	3064	38.6 (34.0–42.0)	6357	** < 0.001**
Haemoglobin (g/L)	133.0 (117.0–146.0)	50,242	133.0 (118.0–146.0)	20,731	135.0 (121.0–148.0)	19,009	127.0 (109.0–142.0)	10,502	** < 0.001**
Interleukin 6 (ng/L)	67.6 (23.0–169.0)	433	43.51 (13.33–89.3)	107	49.4 (21.48–129.5)	128	131.3 (31.1–313.0)	198	** < 0.001**
Lactate dehydrogenase (U/L)	487.0 (349.0–684.0)	7570	445.5 (328.0–621.5)	2966	532.0 (374.0–741.0)	2837	496.0 (353.0–708.5)	1767	** < 0.001**
Lactic acid (mmol/L)	1.5 (1.1–2.1)	21,382	1.4 (1.05–2.0)	9154	1.5 (1.1–2.04)	8553	1.55 (1.1–2.3)	3675	** < 0.001**
Leukocytes (10^9^/L)	8.07 (5.7–12.0)	50,673	7.4 (5.4–10.5)	21,036	7.8 (5.6–11.3)	19,043	10.77 (7.0–18.2)	10,594	** < 0.001**
Lymphocytes (10^9^/L)	0.8 (0.58–1.2)	36,068	0.81 (0.59–1.2)	18,019	0.8 (0.58–1.11)	14,760	0.82 (0.55–1.3)	3289	** < 0.001**
Lymphocytes/leukocytes (%)	9.7 (5.35–15.65)	879	11.0 (6.8–16.1)	357	8.95 (5.0–16.58)	130	8.6 (4.57–15.0)	392	** < 0.001**
Neutrophils (10^9^/L)	5.8 (4.0–8.63)	35,963	5.6 (3.87–8.31)	18,020	5.8 (4.0–8.43)	14,719	7.5 (4.7–11.5)	3224	** < 0.001**
Neutrophils/leukocytes (%)	82.0 (72.9–88.0)	697	81.9 (74.4–87.3)	293	80.4 (70.0–87.7)	101	83.2 (73.2–88.95)	303	0.26
Platelets (10^9^/L)	199.0 (140.0–265.0)	50,263	207.0 (157.0–271.0)	20,811	202.0 (148.0–265.0)	18,903	162.0 (0.3–249.0)	10,549	** < 0.001**
Potassium (mmol/L)	4.1 (3.8–4.5)	35,901	4.1 (3.74–4.5)	14,512	4.1 (3.8–4.5)	12,433	4.2 (3.8–4.6)	8956	** < 0.001**
Procalcitonin (ug/L)	0.24 (0.12–0.7)	6234	0.2 (0.1–0.51)	2191	0.24 (0.12–0.62)	2839	0.4 (0.15–1.38)	1204	** < 0.001**
Prothrombin intl. (ratio)	1.1 (1.02–1.3)	2728	1.09 (1.0–1.2)	611	1.1 (1.03–1.3)	604	1.14 (1.04–1.3)	1513	** < 0.001**
Prothrombin time (s)	13.0 (11.3–14.5)	25,413	12.8 (11.1–14.4)	11,834	13.0 (11.4–14.5)	10,400	13.3 (11.8–14.8)	3179	** < 0.001**
Sodium (mmol/L)	136.0 (133.0–140.0)	38,268	137.0 (134.0–140.0)	15,894	136.0 (133.0–139.0)	13,253	137.0 (133.0–141.0)	9121	** < 0.001**
Troponin I (ug/L)	0.07 (0.02–6.9)	1437	0.03 (0.01–0.25)	398	0.08 (0.02–10.3)	257	0.13 (0.02–10.0)	782	** < 0.001**
Urea nitrogen (mmol/L)	7.7 (5.1–12.85)	46,588	7.1 (4.9–11.5)	19,388	7.3 (5.0–11.6)	17,614	10.35 (6.2–18.56)	9586	** < 0.001**

Bold values indicate statistical significance

*HFNC* high-flow nasal cannula, *NIV* non-invasive mechanical ventilation, *IMV* invasive mechanical ventilation, *IQR* interquartile range, *PTT* partial thromboplastin time

### Patients’ characteristics, in-hospital treatments, and systemic complications

More than 85% of the patients had at least one comorbidity. Hypertension (41.3% [27,521/66,565]) and diabetes mellitus (30.3% [20,164/66,565]) were the most frequently reported comorbid conditions (Table 1). A total of 22.8% [15,190/66,565] of patients were current or past smokers. Complications were also common during the hospital admission (not at hospital presentation), 23.2% [15,470/66,565] developed ARDS, and 20.1% [13,353/66,565] were reported to have an acute renal injury (ARI).

During hospital admission, 61.3% (40,810/66,565) patients received corticosteroid treatment, and 54.6% [36,336/66,565] were admitted to the ICU. Vasopressor/inotrope therapy was used in a quarter of all patients (20.4% [13,592/66,565]), increasing in use according to ventilatory requirement (7.7% [2188/28,256] vs. 17.8% [4282/24,112] vs 50.2 [7122/14,197]). Approximately half of those treated with IMV received vasopressors/inotropes at some point during hospitalisation (50.2% [7122/14,197]). Almost one-quarter of the patients were placed in prone position (23.7% [15,778/66,565]), more commonly in those patients treated with IMV (12.0% [3384/28,256] vs. 27.5% [6628/24,112] vs 40.6% [5766/14,197]). A total of 15.5% [10,287/66,565] of patients failed HFNC/NIV. Moreover, 71.2% [7327/10,287] of the patients that failed HFNC/NIV were registered in HIC and 28.8% [2960/10,287] in LMIC. Finally, 28-day mortality was similar between the different advance ventilatory supports (33.9% [9581/28,256] vs. 38.1% [9177/24,112] vs. 37.1% [5263/14,197]).

### Comparing respiratory support of patients admitted in HIC or LIMC

**Fig. 3:**
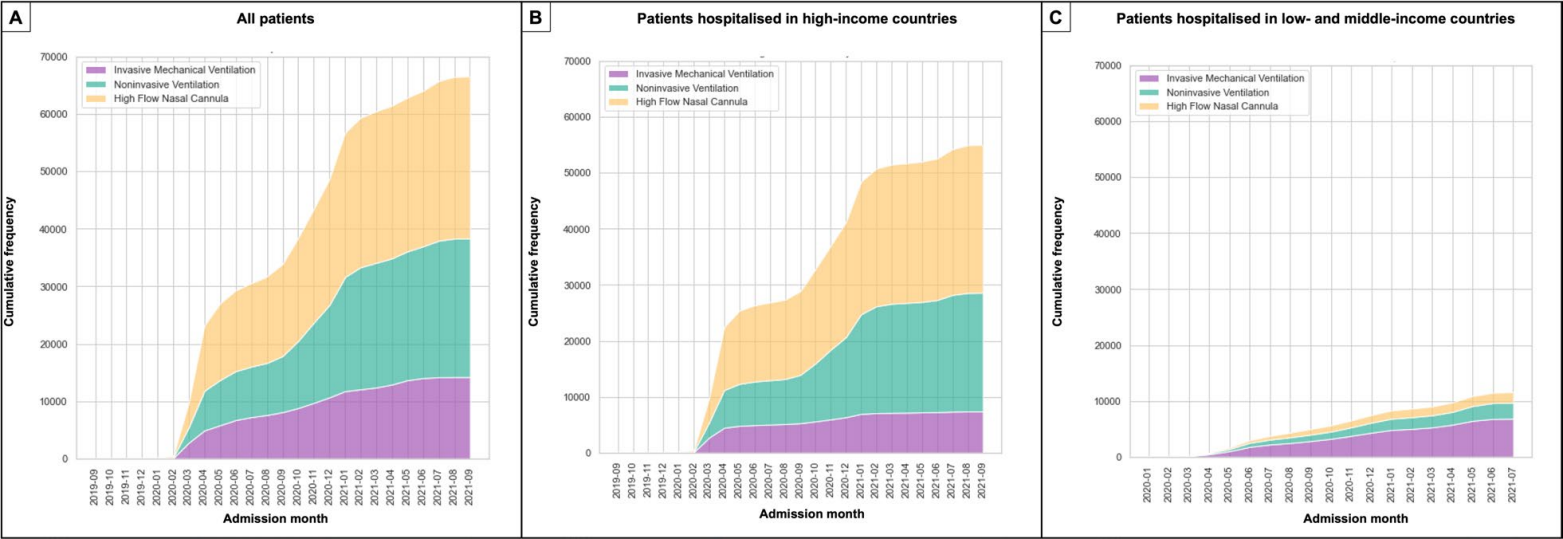
Cumulative frequency (net number of patients) of ventilation treatment given to patients. **A** Complete cohort. **B** Patients from high-income countries. **C** Patients from low middle-income countries

**Fig. 4:**
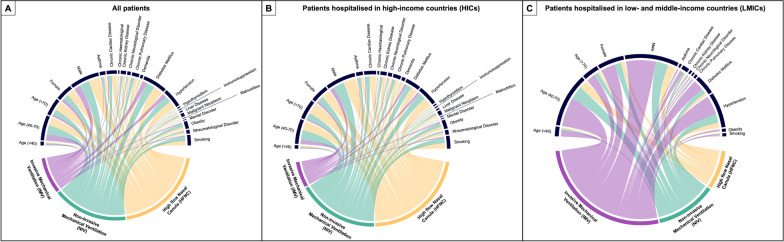
Chord graphic with demographics and comorbidities of patients according to the type of first ventilation treatment received. **A** Complete cohort. **B** Patients from high-income countries. **C** Patients from low middle-income countries

The cumulative frequency of advanced respiratory treatments was stratified by national income classification (Fig. 3). Patients admitted to the hospital in HICs were more frequently treated with HFNC (48.0% [26,399/55,004]), followed by NIV (38.6% [21,237/55,004]) and IMV (13.4% [7368/55,004]). In contrast, patients admitted in LMICs were less frequently treated with HFNC (16.1% [1857/11,561]), and the majority received IMV (59.1% [6829/11,561]) (Table 1; Fig. 3). We also found differences in distribution among the different types of respiratory support when stratified by income classification and respiratory support (Fig. 4).

Patients treated with IMV in HICs had fewer comorbid conditions and were more frequently between 40 and 70 years old. In sharp contrast, patients in LMIC who were younger than 40 years old often received IMV and were more frequently male. Also, they were mostly treated with IMV rather than non-invasive respiratory strategies (Fig. 4).

### Changes in respiratory supports

**Fig. 5:**
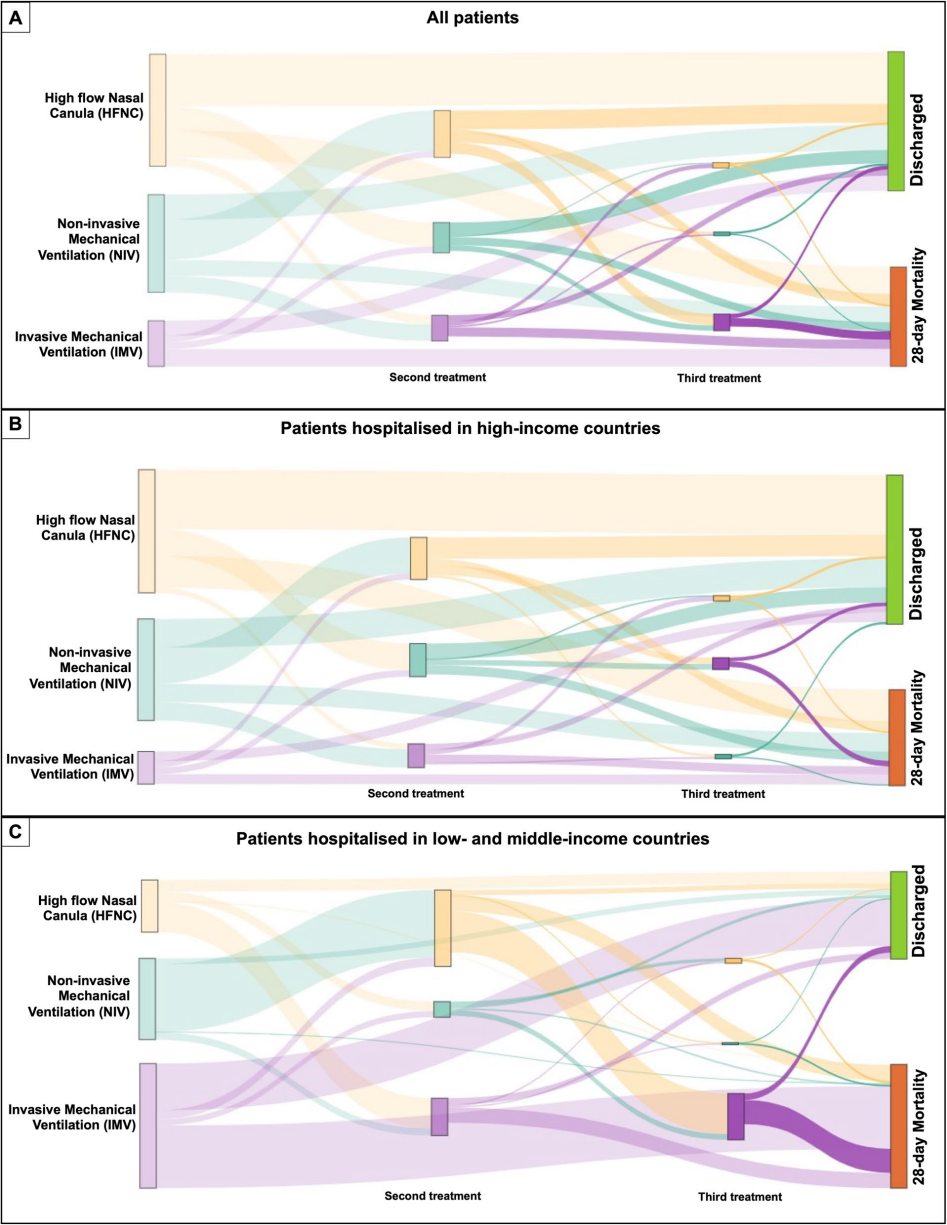
Alluvia diagram of the patients’ transitions between ventilation treatments and clinical outcomes. The width of the links is proportional to the number of patients. **A** Complete cohort. **B** Patients from high-income countries. **C** Patients from low middle-income countries

Figure 5 presents the alluvia diagrams illustrating how patients progressed among respiratory support during hospital admission. Notably, patients who required more than one respiratory treatment had higher mortality than those treated with only one type of support, whether the first respiratory support was HFNC, NIV, or IMV (Fig. 5).

### Risk factors for failing HFNC or NIV as first respiratory support

**Fig. 6:**
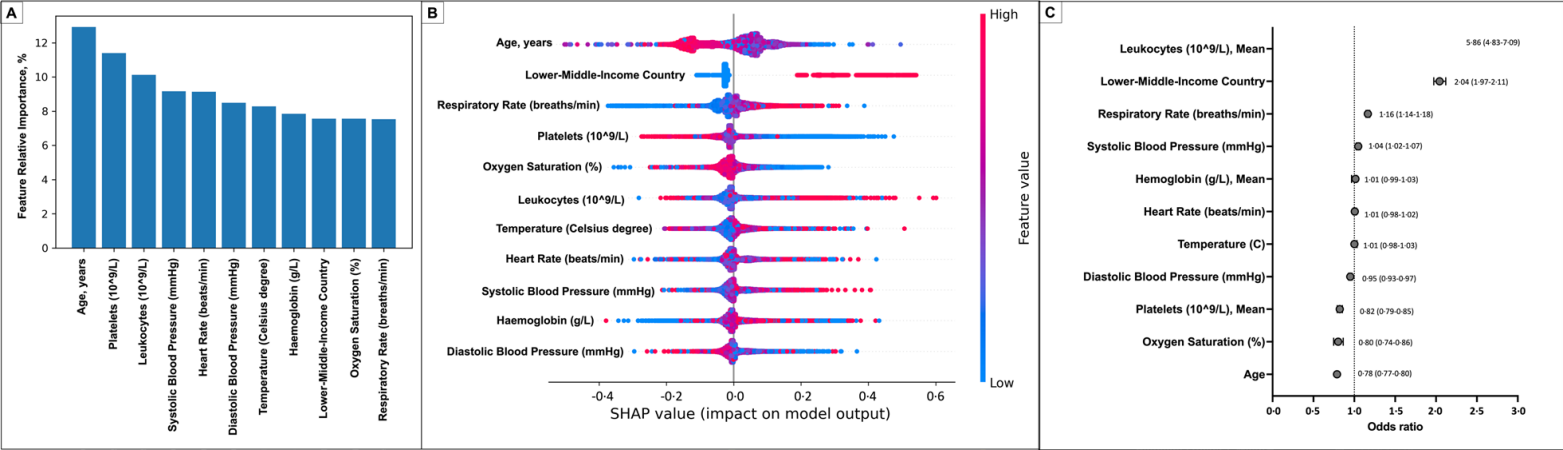
An automatised model to determine risk factors associated with non-invasive ventilation failure. **A** variables more strongly related to non-invasive ventilation failure according to the Gini importance. **B** the contribution of the variables to the output; the red values indicate a high-value contribution of the variable, and the blue values a low-value contribution. The positive values in the plot indicate a high probability of 28-day fatality, and negative values indicate a low likelihood of 28-day fatality. Panel C presents a logistic regression model, showing variables more strongly associated with the 28-day fatality ratio. The most significant variables were leucocyte count, low-/middle-income country attention, higher respiratory rate, and higher systolic blood pressure

The failure rate of HFNC or NIV was 15.5% [10,287/66,565]. According to the Gini importance, the variables most strongly associated with non-invasive ventilation failure (either HFNC or NIV) were age, lower platelets, and higher leukocyte count during the first 24 h of hospital admission (Fig. 6A). In the logistic regression analysis, we found that high leukocyte counts at hospital admission (OR [95% CI]; 5.86 [4.83–7.10]), treatment in an LMIC (OR [95% CI]; 2.04 [1.97–2.11]), and tachypnoea at hospital admission (OR [95% CI]; 1.16 [1.14–1.18]) were strongly associated factors with IMV treatment as rescue treatment (Fig. 6B, C).

### Clinical outcomes and risk factors associated with 28-day fatality ratio

**Fig. 7:**
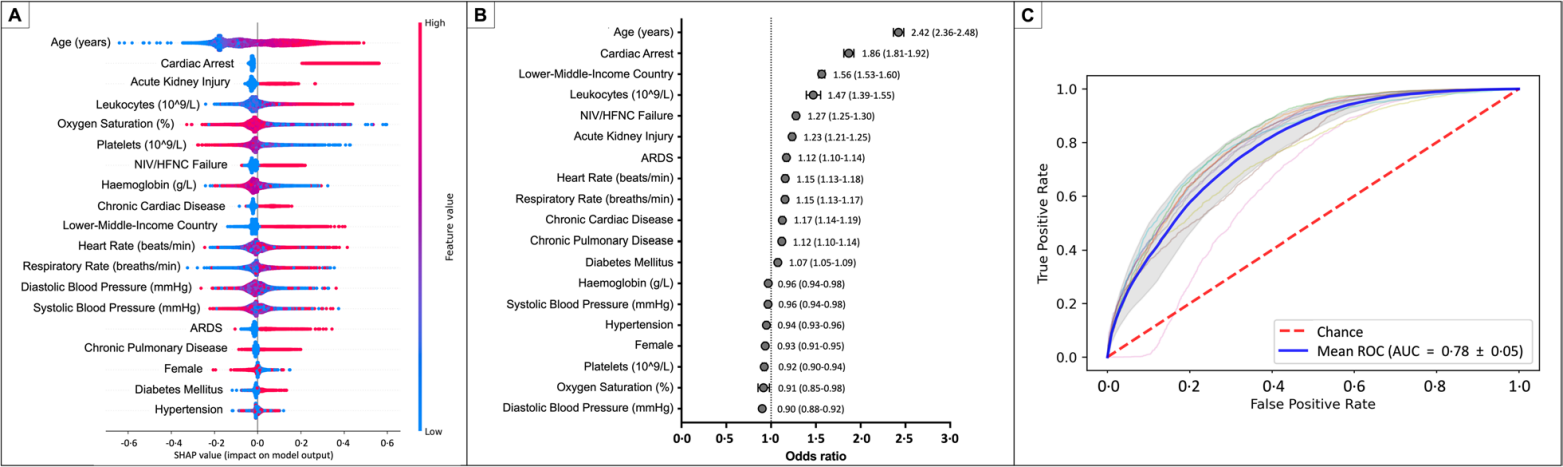
An automatised model to determine risk factors associated with the 28-day fatality ratio. **A** The contribution of the variables to the output; the red values indicate a high-value contribution of the variable, and the blue values a low-value contribution. The positive values in the plot indicate a high probability of 28 fatalities, and negative values indicate a low likelihood of 28-day fatality. **B** A logistic regression model, showing variables more strongly associated with the 28-day fatality ratio. **C** Each cross-validation trial's receiver operative curve (ROC) for the subset of the selected variables. The blue curve represents the average of the ROC curves of each test, and the average area under the ROC is also presented. The most significant variables associated with the 28-day fatality ratio were age, cardiac arrest, low-/middle-income country attention, and leucocyte count. Also, patients that fail the non-invasive or high-flow nasal cannula are independently associated with a higher 28-day fatality ratio

Almost half of the patients treated with HFNC [46.3%; 11,954/28,256] and 37.1% (5263/14,197) of patients treated with IMV died within 28 days. The variables identified as risk factors associated with the 28-day fatality ratio are shown in Fig. 7. Older age (OR [95% CI]; 2.42 [2.36–2.48]), cardiac arrest during hospitalisation (OR [95% CI]; 1.86 [1.81–1.92]), receiving treatment in an LMIC (OR [95% CI]; 1.56 [1.53–1.60]), and higher leukocyte counts at hospital admission (OR [95% CI]; 1.47 [1.39–1.55]) were the main adjusted risk factors associated with 28-day mortality. Notably, NIV/HFNC failure (OR [95% CI]; 1.27 [1.25–1.30]) was also highly associated with fatality. Other factors were acute kidney injury (OR [95% CI]; 1.23 [1.21–1.25]), ARDS (diagnosed during the hospital admission, not during the first 24 h) (OR [95% CI]; 1.12 [1.10–1.14]), increased heart rate at admission (OR [95% CI]; 1.15 [1.13–1.18]), increased respiratory rate at admission (OR [95% CI]; 1.15 [1.13–1.17]), chronic cardiac diseases (OR [95% CI]; 1.17 [1.14–1.19]), chronic pulmonary diseases (OR [95% CI]; 1.12 [1.10–1.14]), and diabetes mellitus (OR [95% CI]; 1.07 [1.05–1.09]). The model used to predict the 28-day fatality ratio had a good discriminatory capacity when evaluated by the AUROC (mean [SD] 0.78 [0.05], Fig. 7).

## Discussion

In this large, multinational, prospective cohort study, we found that patients with severe COVID-19 were mainly treated with non-invasive respiratory strategies (i.e. HNFC or NIV) in HICs; in contrast, patients with severe COVID-19 in LMICs were more frequently treated with IMV. We found that the 28-day fatality ratio was similar among patients treated with HFNC, NIV, or IMV worldwide. Notably, we found that patients treated with IMV as rescue therapy (i.e. failure of non-invasive treatments) had a higher 28-day fatality ratio than patients treated with IMV earlier in their disease course. The risk factors associated with failing the non-invasive respiratory strategies were high leukocyte counts at admission, increased heart rate at admission, and being treated in an LMIC. Notably, being admitted during the first pandemic wave did not impact clinical outcomes or respiratory treatments.

Early in the pandemic, healthcare workers identified that patients with hypoxemia could be treated with HFNC [21–23]. International guidelines also recommend non-invasive respiratory support as the first treatment, and many centres utilise HFNCs outside formal ICU settings [13]. Notably, the widespread usage of HFNC and NIV in patients with severe COVID-19 was recommended by experts and guidelines but not supported by high-quality data. Later, Ospina-Tascon et al. [12] carried out a multicentre, open randomised clinical trial and found that the early treatment with HFNC compared to conventional oxygen treatment was associated with a lower necessity of IMV (34.3 Vs 51.0, HR: 1.39; 95% CI 1.00–1.92; *p* = 0.04). Then, Perkins et al. [24] in the RECOVERY-RS trial found that NIV was associated with a lower requirement of tracheal intubation and lower 30-day mortality when compared to conventional oxygen therapy (absolute difference, − 8% [95% CI, − 15 to − 1%], *p* = 0.03). Our study found that HFNC, NIV, and IMV have similar 28-day fatality ratios, in concordance with prior literature. However, we found that HFNC was mainly used in HIC, which might be in relation to the capacity of these countries to acquire this new technology during the pandemic and the ability of these countries to expand their bed capacity to treat patients with HFNC outside of the ICU. Also, some patients or their families do not accept endotracheal intubation and prefer non-invasive strategies, though our study did not collect these data.

In contrast to HIC, the most common respiratory treatment in patients with severe COVID-19 utilised in LMIC has been IMV, as is evident in our data. Estenssoro et al. [17] described the results of a prospective observational cohort of patients admitted to 64 ICUs in Argentina. They included 1909 patients treated with IMV and found that lung-protective respiratory strategies were widely used but with a high fatality rate among patients included in the cohort (57.7%, 1101/1909). In another study in Brazil, Ranzani et al. [7] found that 23% (45,205/232,036) of patients admitted to the hospital were treated with IMV. They also found that the fatality rate among those receiving IMV was 80% during the first pandemic wave and 87% during the second wave [7, 25]. Notably, they found that 14% (5976/44,055) of the patients treated with IMV were treated outside of the ICU [25]. These results highlight that fatality rates and treatments changed during the pandemic and differed for each country. Moreover, these data align with our results, showing that IMV was frequently used in LMIC and that many patients with severe COVID-19 were treated outside of ICU [8, 26]. Notably, the impact of ICU admission on clinical outcomes was already explored in our cohort and published elsewhere [3]. We found that ICU admission was associated with better clinical outcomes independently of disease severity, treatments received, income classification, and system saturation (i.e. the number of new COVID-19 detected the day patients was admitted).

Even though non-invasive respiratory support has been proven effective in treating patients with severe hypoxemia during COVID-19, up to 30% of the patients were treated with IMV as a rescue treatment. Thus, it is essential to identify which patients might be at risk of failing under the non-invasive respiratory strategy and not to delay IMV in these patients. Rodriguez A. et al*.,* in one of the largest prospective cohorts of patients admitted to the ICU due to severe flu infection, found that patients who failed NIV had a mortality rate three times higher than those who did not fail [27]. Also, they found that patients who failed NIV had higher mortality than those treated with IMV as initial treatment (38.4 vs 31.3, *p* = 0.18). In a multicentre COVID-19 study, Boscolo A. et al*.* found that 704 patients who failed non-invasive respiratory support had an accumulative fatality rate of 43% [28]. Our findings support that patients with severe COVID-19 who fail the initial respiratory support with non-invasive treatments have a higher mortality rate and were independently associated with 28-day fatality. Also, we found that patients with higher leukocyte counts at admission, higher respiratory rate at admission, and being in an LMIC were at higher risk of failing the non-invasive respiratory strategies. Thus, patients with these characteristics should be carefully evaluated to avoid delays in initiating IMV when appropriate.

Our study has strengths and limitations that are important to acknowledge. First, the respiratory support interventions were not according to a standardised protocol, leaving clinical teams to choose when to use HFNC, NIV, or IMV; thus, demographic or clinical characteristics may differ across the groups studied. However, we performed a robust statistical analysis using random forest analyses and logistic regression, adjusting for several confounders. This allowed us to evaluate linear and nonlinear relations in a supervised statistical approach. Second, most patients in our study were registered in Europe and HICs, which might constitute a significant selection bias. However, we had more than 11,000 patients in LMICs in Africa, South America, and Asia, including a large cohort of patients and contributing to our results' global generalisability. Third, we do not have complete data on specific respiratory parameters used during the support (i.e. peep, flows, FiO_2_, volumes, among many others), limiting our capacity to assess the rates of protective respiratory strategies, among other essential factors. Thus, these results cannot imply a causal association between respiratory support device treatments and clinical outcomes. Each patient should be evaluated carefully with decisions on the type of respiratory support based upon the evolving evidence base applied to their specific clinical condition and goals of care. Finally, throughout the COVID-19 pandemic, patients were treated with a large variety of medications and supportive clinical protocols; it is challenging to make conclusions about the factors associated with 28-day fatality using observational study methodologies in such a dynamic context.

## Conclusions

Patients hospitalised with confirmed COVID-19 are often treated with advanced respiratory support. HFNC was the primary initial respiratory support used during the pandemic; however, this treatment was mainly used in HIC. In contrast, IMV was the primary respiratory treatment utilised in LMIC. Non-invasive respiratory treatments (i.e. HFNC and NIV) could be used as the first respiratory support in patients with severe COVID-19; however, it is crucial to identify patients at risk of failing because delaying IMV may be associated with worse clinical outcomes. Further studies are needed to confirm these associations.

## Supplementary Information

**Additional file 1.** Supplemental methods.

**Additional file 2.** Conflict of interests.

## Data Availability

The datasets used and/or analysed during the current study are available in the Infectious Diseases Data Observatory (IDDO, www.iddo.org).

## Abbreviations

ICU: Intensive care unit
VA-LRTI: Ventilator-associated lower respiratory tract infection
COVID-19: Coronavirus disease-19
HIV/AIDS: Human immunodeficiency virus/acquired immunodeficiency syndrome
SARS-CoV-2: Severe Respiratory Syndrome Coronavirus 2
VAP: Ventilator-associated pneumonia
VAT: Ventilator-associated tracheobronchitis
rt-PCR: Reverse transcription-polymerase chain reaction
IMV: Invasive mechanical ventilation
ERS: European Respiratory Society
ESICM: European Society of Intensive Care Medicine
ESCMID: European Society of Clinical Microbiology and Infectious Diseases
ALAT: Asociación Latinoamericana del Tórax
ETA: Endotracheal aspirates
LOS: Length of stay
RF: Random forest
AUROC: Area under the model's receiver operating curve
ORs: Odds ratios
IQR: Interquartile range
CRP: C reactive protein
AKI: Acute renal injury
HRs: Hazard ratio

## References

[1] Akbar S, Pan D, Ehdode A, Islam R, Abouzaid A, Balasundaram K, Shihadeh M, Patel K, Othman G, Umerah O (2022). Prognostic value of maximum NEWS-2 scores in addition to ISARIC 4C scores for patients admitted to hospital with COVID-19. J Infect.

[2] Knight SR, Gupta RK, Ho A, Pius R, Buchan I, Carson G, Drake TM, Dunning J, Fairfield CJ, Gamble C (2022). Prospective validation of the 4C prognostic models for adults hospitalised with COVID-19 using the ISARIC WHO Clinical Characterisation Protocol. Thorax.

[3] Reyes LF, Murthy S, Garcia-Gallo E, Irvine M, Merson L, Martin-Loeches I, Rello J, Taccone FS, Fowler RA, Docherty AB (2022). Clinical characteristics, risk factors and outcomes in patients with severe COVID-19 registered in the International Severe Acute Respiratory and Emerging Infection Consortium WHO clinical characterisation protocol: a prospective, multinational, multicentre, observational study. ERJ Open Res.

[4] Knight SR, Ho A, Pius R, Buchan I, Carson G, Drake TM, Dunning J, Fairfield CJ, Gamble C, Green CA (2020). Risk stratification of patients admitted to hospital with covid-19 using the ISARIC WHO Clinical Characterisation Protocol: development and validation of the 4C Mortality Score. BMJ.

[5] Network C-IGobotR, the C-ICUI: Clinical characteristics and day-90 outcomes of 4244 critically ill adults with COVID-19: a prospective cohort study. Intensive Care Med. 2021;47(1):60–73. 10.1007/s00134-020-06294-x PMC7674575 33211135

[6] Grasselli G, Zangrillo A, Zanella A, Antonelli M, Cabrini L, Castelli A, Cereda D, Coluccello A, Foti G, Fumagalli R (2020). Baseline characteristics and outcomes of 1591 patients infected with SARS-CoV-2 admitted to ICUs of the Lombardy Region, Italy. JAMA.

[7] Ranzani OT, Bastos LSL, Gelli JGM, Marchesi JF, Baiao F, Hamacher S, Bozza FA (2021). Characterisation of the first 250,000 hospital admissions for COVID-19 in Brazil: a retrospective analysis of nationwide data. Lancet Respir Med.

[8] Reyes LF, Bastidas A, Narvaez PO, Parra-Tanoux D, Fuentes YV, Serrano-Mayorga CC, Ortiz V, Caceres EL, Ospina-Tascon G, Diaz AM (2022). Clinical characteristics, systemic complications, and in-hospital outcomes for patients with COVID-19 in Latin America. LIVEN-Covid-19 study: a prospective, multicenter, multinational, cohort study. PLoS ONE.

[9] Quah P, Li A, Phua J (2020). Mortality rates of patients with COVID-19 in the intensive care unit: a systematic review of the emerging literature. Crit Care.

[10] Rodriguez A, Ruiz-Botella M, Martin-Loeches I, Jimenez Herrera M, Sole-Violan J, Gomez J, Bodi M, Trefler S, Papiol E, Diaz E (2021). Deploying unsupervised clustering analysis to derive clinical phenotypes and risk factors associated with mortality risk in 2022 critically ill patients with COVID-19 in Spain. Crit Care.

[11] Greco M, De Corte T, Ercole A, Antonelli M, Azoulay E, Citerio G, Morris AC, De Pascale G, Duska F, Elbers P (2022). Clinical and organizational factors associated with mortality during the peak of first COVID-19 wave: the global UNITE-COVID study. Intensive Care Med.

[12] Ospina-Tascon GA, Calderon-Tapia LE, Garcia AF, Zarama V, Gomez-Alvarez F, Alvarez-Saa T, Pardo-Otalvaro S, Bautista-Rincon DF, Vargas MP, Aldana-Diaz JL (2021). Effect of high-flow oxygen therapy vs conventional oxygen therapy on invasive mechanical ventilation and clinical recovery in patients with severe COVID-19: a randomized clinical trial. JAMA.

[13] Chalmers JD, Crichton ML, Goeminne PC, Cao B, Humbert M, Shteinberg M, Antoniou KM, Ulrik CS, Parks H, Wang C (2021). Management of hospitalised adults with coronavirus disease 2019 (COVID-19): a European Respiratory Society living guideline. Eur Respir J.

[14] Dondorp AM, Hayat M, Aryal D, Beane A, Schultz MJ (2020). Respiratory support in COVID-19 patients, with a focus on resource-limited settings. Am J Trop Med Hyg.

[15] Alqahtani JS, Mendes RG, Aldhahir A, Rowley D, AlAhmari MD, Ntoumenopoulos G, Alghamdi SM, Sreedharan JK, Aldabayan YS, Oyelade T (2020). Global current practices of ventilatory support management in COVID-19 patients: an international survey. J Multidiscip Healthc.

[16] Weerakkody S, Arina P, Glenister J, Cottrell S, Boscaini-Gilroy G, Singer M, Montgomery HE (2022). Non-invasive respiratory support in the management of acute COVID-19 pneumonia: considerations for clinical practice and priorities for research. Lancet Respir Med.

[17] Estenssoro E, Loudet CI, Rios FG, Kanoore Edul VS, Plotnikow G, Andrian M, Romero I, Piezny D, Bezzi M, Mandich V (2021). Clinical characteristics and outcomes of invasively ventilated patients with COVID-19 in Argentina (SATICOVID): a prospective, multicentre cohort study. Lancet Respir Med.

[18] ISARIC Clinical Characterisation Group (2021). COVID-19 symptoms at hospital admission vary with age and sex: results from the ISARIC prospective multinational observational study. Infection.

[19] Garcia-Gallo E, Merson L, Kennon K, Kelly S, Citarella BW, Fryer DV, Shrapnel S, Lee J, Duque S, ISARIC Clinical Characterisation Group (2022). ISARIC-COVID-19 dataset: a prospective, standardized, global dataset of patients hospitalized with COVID-19. Sci Data.

[20] Harris PA, Taylor R, Thielke R, Payne J, Gonzalez N, Conde JG (2009). Research electronic data capture (REDCap)—a metadata-driven methodology and workflow process for providing translational research informatics support. J Biomed Inform.

[21] Li J, Fink JB, Ehrmann S (2020). High-flow nasal cannula for COVID-19 patients: low risk of bio-aerosol dispersion. Eur Respir J.

[22] Wang K, Zhao W, Li J, Shu W, Duan J (2020). The experience of high-flow nasal cannula in hospitalized patients with 2019 novel coronavirus-infected pneumonia in two hospitals of Chongqing, China. Ann Intensive Care.

[23] Arentz M, Yim E, Klaff L, Lokhandwala S, Riedo FX, Chong M, Lee M (2020). Characteristics and outcomes of 21 critically ill patients with COVID-19 in Washington State. JAMA.

[24] Perkins GD, Ji C, Connolly BA, Couper K, Lall R, Baillie JK, Bradley JM, Dark P, Dave C, De Soyza A (2022). Effect of noninvasive respiratory strategies on intubation or mortality among patients with acute hypoxemic respiratory failure and COVID-19: the RECOVERY-RS randomized clinical trial. JAMA.

[25] Bastos LS, Ranzani OT, Souza TML, Hamacher S, Bozza FA (2021). COVID-19 hospital admissions: Brazil's first and second waves compared. Lancet Respir Med.

[26] Reyes LF, Rodriguez A, Bastidas A, Parra-Tanoux D, Fuentes YV, Garcia-Gallo E, Moreno G, Ospina-Tascon G, Hernandez G, Silva E (2022). Dexamethasone as risk-factor for ICU-acquired respiratory tract infections in severe COVID-19. J Crit Care.

[27] Rodriguez A, Ferri C, Martin-Loeches I, Diaz E, Masclans JR, Gordo F, Sole-Violan J, Bodi M, Aviles-Jurado FX, Trefler S (2017). Risk factors for noninvasive ventilation failure in critically ill subjects with confirmed influenza infection. Respir Care.

[28] Boscolo A, Pasin L, Sella N, Pretto C, Tocco M, Tamburini E, Rosi P, Polati E, Donadello K, Gottin L (2021). Outcomes of COVID-19 patients intubated after failure of non-invasive ventilation: a multicenter observational study. Sci Rep.

