# Supplementary material for: Respiratory support in patients with severe COVID-19 in the International Severe Acute Respiratory and Emerging Infection (ISARIC) COVID-19 study: a prospective, multinational, observational study
Source: Crit Care. 2022 Sep 13;26:276. doi: 10.1186/s13054-022-04155-1 (PMC9469080; doi:10.1186/s13054-022-04155-1)
Supplement: Supplementary file 1 — Additional file 1. Supplemental methods. [file 13054_2022_4155_MOESM1_ESM.docx]

**Abstract word count:** 300

**Manuscript word count:** 3374

**Abbreviated running title:** Respiratory Support in a COVID-19 Cohort Stratified by country's income.

**TITLE:** Respiratory Support in Patients with Severe COVID-19 in the ISARIC Covid-19 Study: A Prospective, Multinational, Observational Study.

**AUTHORS:** The ISARIC Characterization Group^1^*, Luis Felipe Reyes^2,3,1#^, Srinivas Murthy^4^, Esteban Garcia-Gallo^2^, Laura Merson^1^, Elsa D. Ibañez^2^, Jordi Rello^5^, Yuli Viviana Fuentes^1,2^, Ignacio Martin-Loeches^6^, Fernando Bozza^7^, Sara Duque^1^, Fabio S. Taccone^8^, Robert A. Fowler^9^, Christiana Kartsonaki^1^, Bronner P. Gonçalves^1^, Barbara Wanjiru Citarella^1^, Diptesh Aryal^10^, Erlina Burhan^11^, Matthew J. Cummings^12^, Christelle Delmas^13^, Rodrigo Diaz^14^, Claudia Figueiredo-Mello^15^, Madiha Hashmi^16^, Prasan Kumar Panda^17^, Miguel Pedrera Jiménez^18^, Diego Fernando Bautista Rincon^19^, David Thomson^20^, Alistair Nichol^21^, Jhon Marshall^22^ and Piero L. Olliaro^1^.

*****The Complete list of authors and affiliations is listed at the end of the manuscript.

**Affiliations:** 1, Nuffield School of Medicine, University of Oxford, Oxford, United Kingdom; 2, Infectious Diseases Department, Universidad de La Sabana, Chía, Colombia; 3, Critical Care Department, Clínica Universidad de La Sabana, Chía, Colombia; 4, Department of Pediatrics, University of British Columbia, Vancouver, Canada; 5, Clinical Research/Epidemiology in Pneumonia & Sepsis (CRIPS), Vall d'Hebron Institute of Research (VHIR), Barcelona, Spain; Centro de Investigación Biomédica En Red de Enfermedades Respiratorias (CIBERES), Instituto de Salud Carlos III, Madrid, Spain; 6, Department of Clinical Medicine, St James's Hospital, Multidisciplinary Intensive Care Research Organization (MICRO), Dublin, Ireland; 7, D'Or Institute for Research and Education (IDOR), Rio de Janeiro, RJ, Brazil; Brazilian Research in Intensive Care Network (BRICNet), Brazil; Oswaldo Cruz Foundation (FIOCRUZ), Rio de Janeiro, RJ, Brazil; 8, Department of Intensive Care, Université Libre de Bruxelles (ULB) and Laboratoire de Recherche Experimentale, Department of Intensive Care, Hôpital Erasme, Brussels, Belgium; 9; Interdepartmental Division of Critical Care Medicine, University of Toronto, Toronto, ON, Canada; 10, ; 11, ; 12, Division of Pulmonary, Allergy, and Critical Care Medicine, Department of Medicine, Columbia University Vagelos College of Physicians and Surgeons, New York, NY, USA ; 13, ; 14, ; 15 Instituto de Infectologia Emílio Ribas, São Paulo, Brazil, ; 16, ; 17, ; 18, ; 19, ; 20, ; 21, University College Dublin Clinical Research Centre at St Vincent's University Hospital, Dublin, Ireland; 22, Li Ka Shing Knowledge Institute, Unity Health Toronto, St Michael's Hospital, Toronto, Ontario, Canada.

**#Author for Correspondence:** Luis Felipe Reyes, MD., PhD., Universidad de La Sabana, Chía, Colombia. Phone number: 57 861 55 55 Ext: 23342. Email: [luis.reyes5@unisabana.edu.co](mailto:luis.reyes5@unisabana.edu.co)

**Potential Conflict of interest:** Attached.

**ONLINE SUPPLEMENT**

**METHODS:**

The Gini importance is based on the Gini impurity, which measures how well a potential split of a decision tree separates the samples of the two classes in a particular node; this metric is computed exhaustively over the random subset of variables used by the RF, for each specific decision tree, allowing to identify the optimal split (the feature and threshold that gives the purest nodes). The decrease in Gini impurity resulting from this optimal split is recorded individually and accumulated for all variables in all the nodes in all the decision trees in the random forest; this quantity is known as the Gini importance and indicates how often a particular feature was selected for a split, and how large its overall discriminative value was for the classification problem under study. In general, the higher the Gini importance, the more important the feature, and it was used for variable selection. Subsequently, to interpret the contribution of the optimal subset of variables to the model, the Python Treeinterpreter library was used. The library contains functions that allow decomposing each prediction into bias and feature contribution components, as follows: prediction = bias + feature_1_contribution + ... + feature_n_contribution. With this tool, the random forest model can be interpreted by quantifying the contribution of each variable to predicting the outcome (i.e., 28-day fatality ratio or non-invasive respiratory support failure).
