# Supplementary material for: Respiratory support in patients with severe COVID-19 in the International Severe Acute Respiratory and Emerging Infection (ISARIC) COVID-19 study: a prospective, multinational, observational study
Source: Crit Care. 2022 Sep 13;26:276. doi: 10.1186/s13054-022-04155-1 (PMC9469080; doi:10.1186/s13054-022-04155-1)
Supplement: Supplementary file 2 — Additional file 2. Conflict of interests. [file 13054_2022_4155_MOESM2_ESM.docx]

**Potential Conflicts of Interest**

Allavena, C. declares personal fees from ViiVHealthcare, MSD, Janssen, and Gilead, all outside the submitted work. Andréjak, C. declares personal fees for lectures from Astra Zeneca, outside the submitted work. Antonelli, M. declares unrestricted research grants from GE and Estor/Toray, Board participation from Pfizer and Shionogi. All unrelated to the present work. Beltrame, A. has nothing to declare concerning the current work. A Borie, R. declares personal fees for Roche, Sanofi, and Boehringer Ingelheim's lectures outside the submitted work. Bosse, Hans Martin is co-investigator for placebo studies in infants and children in clinical trials by Actelion/Janssen (Johnson&Johnson), outside the submitted work. Cheng, M. declares grants from McGill Interdisciplinary Initiative in Infection and Immunity, grants from Canadian Institutes of Health Research, during the conduct of the study; personal fees from GEn1E Lifesciences (as a member of the scientific advisory board), personal fees from nplex biosciences (as a member of the scientific advisory board), outside the submitted work. He is the co-founder of Kanvas Biosciences and owns equity in the company. In addition, M. Cheng reports a patent Methods for detecting tissue damage, graft versus host disease, and infections using cell-free DNA profiling pending, and a patent Methods for assessing the severity and progression of SARS-CoV-2 infections using cell-free DNA pending. Cholley, B. declares personal fees (for lectures and participation to advisory boards) from Edwards, Amomed, Nordic Pharma, and Orion Pharma. Claure-Del Granado, R. declares individual fees (for lectures and participation to advisory boards) from Nova Biomedical, Medtronic, and Baxter all outside the submitted work. Cruz-Bermúdez J.L. declares personal fees from Elsevier for advice outside the submitted work. Cummings, M. and O’Donnell, M. participated as investigators for clinical trials evaluating the efficacy and safety of remdesivir (sponsored by Gilead Sciences) and convalescent plasma (sponsored by Amazon) in hospitalized patients with COVID-19. Support for this work is paid to Columbia University. Dalton, H. declares personal fees for medical director of Innovative ECMO Concepts and honorarium from Abiomed/BREETHE Oxi-1 and Instrumentation Labs. Consultant fee, Entegrion Inc.

Dyrhol-Riise, AM, declares grants from Gilead outside this work. Deplanque, D. declares personal fees from Biocodex, Bristol-Myers Squibb, and Pfizer (advisory boards). Donnelly, C.A. declares research funding from the UK Medical Research Council and the UK National Institute for Health Research. Douglas, J.J.  declares personal fees from lectures from Sunovion and Merck, consulting fees from Pfizer. Durante-Mangoni, E. declares funding via his Institution from MSD, Pfizer, and personal fees or participation in advisory boards or participation to the speaker’s bureau of Roche, Pfizer, MSD, Angelini, Correvio, Nordic Pharma, Bio-Merieux, Abbvie, Sanofi-Aventis, Medtronic, Tyrx and DiaSorin. Gordon, AC. reports a Research Professorship from NIHR (RP-2015-06-18), consulting fees from GlaxoSmithKline, Bristol Myers Squibb, and 30 Respiratory paid to his institution outside of the submitted work. Grasselli, G. declares personal fees from Getinge, Biotest, Draeger Medical, Fisher & Paykel, MSD, and unrestricted research grants from MSD and Fisher & Paykel, all outside the submitted work. Guerguerian AM. Participated as a site investigator for the Hospital for Sick Children, Toronto, Canada, through the SPRINT-SARI Study via the Canadian Critical Care Trials Group sponsored in part by the Canadian Institutes of Health Research. Hammond, TC declares consulting fees from Regeneron, Pfizer, and Agenus. Ho, A. declares grant funding from Medical Research Council UK, Scottish Funding Council - Grand Challenges Research Fund, and the Wellcome Trust, outside this submitted work. Holter, J. C. reports grants from Research Council of Norway grant no 312780, and Vivaldi Invest A/S owned by Jon Stephenson von Tetzchner, during the conduct of the study. Hulot, J.S. reports grants from Bioserenity, Sanofi, Servier, and Novo Nordisk.; speaker, advisory board or consultancy fees from Amgen, Astra Zeneca, Bayer, Bioserenity, Boehringer Ingelheim, Bristol-Myers Squibb, MSD, Novartis, Novo Nordisk, Vifor (all unrelated to the present work). Kalleberg, K.T. Is a founder and shareholder of the company Age Labs, which develops epigenetic tests, including one for COVID-19 severity. Kimmoun, A. declares personal fees (payment for lectures) from Baxter, Aguettant, Aspen. Kumar, D. declares grants and personal fees from Roche, GSK, and Merck, and personal fees from Pfizer and Sanofi. Kutsogiannis, D.J. declares personal fees for a lecture from Tabuk Pharmaceuticals and the Saudi Critical Care Society. Kutsyna, G. declares the study consulting fee for clinical trial ClinicalTrials.gov Identifier: NCT04762628. Laffey, J. reports that he has received fees for consultancy from GlaxoSmithKline and from Baxter Therapeutics for work outside the scope of this work. Lairez, O. declares grant funding from Pfizer; conference fees from Amicus, GE Healthcare, Novartis, Sanofi-Genzyme, and Takeda-Shire; and consultancy fees from Alnylam, Amicus, Pfizer, Takeda-Shire. Lee, J. reports grants from European Commission PREPARE grant agreement No 602525, European Commission RECOVER Grant Agreement No 101003589 and European Commission ECRAID-Plan Grant Agreement 825715 supporting the conduct, coordination, and management of the work. Lee, T.C. declares research salary support from les Fonds de recherche du Québec – Santé. Lefèvre, B. declares travel/accommodation/meeting expenses from Mylan and Gilead, all outside the submitted work. Lellouche, F. declares grants from CIHR for COVID-19 studies is co-founder and administrator of Oxynov. inc, fees from Fisher&Paykel, Vygon, and Novus. Lemaignen, A. declares personal fees (payment for lectures) from MSD and Gilead; and travel/accommodation/meeting expenses from Pfizer. Leone, M declares personal fees from Gilead, MSD, Aspen, Ambu, and Amomed Lescure, F.X. declares personal fees (payment for lectures) from Gilead, MSD; and travel/accommodation/meeting expenses from Astellas, Eumedica, MSD. Lim, W.S. declares his institution has received unrestricted investigator-initiated research funding from Pfizer for an unrelated multicentre cohort study in which he is the Chief Investigator, and research funding from the National Institute for Health Research, the UK, for various clinical trials outside the submitted work. Liu, K. reports personal fees from MERA and receives a salary from TXP Medical completely outside the submitted work. Maier, Lars S. has nothing to declare with respect to the present work. Mark, P.B. declares grant support from Boehringer Ingelheim, lecture fees and/or travel support from AstraZeneca, Astellas, Vifor, Pharmacosmos, and Napp, and grant funding from British Heart Foundation, Medical Research Council, National Institute for Health Research and Kidney Research UK all outside the submitted work. Martin-Blondel G declares support for attending meetings and personal fees from BMS, MSD, Janssen, Sanofi, Pfizer, and Gilead for lectures outside the submitted work. Martin-Loeches I. declared lectures for Gilead, Thermofisher, Pfizer, MSD; advisory board participation for Fresenius Kabi, Advanz Pharma, Gilead, Accelerate, Merck; and consulting fees for Gilead outside of the submitted work. Martín-Quiros, A. declares consulting fees for Gilead. Mentré F declares consulting fees from IPSEN, Servier, and Da Volterra and reports research grants to her group from Sanofi, Roche, Servier, and Da Voleterra, all outside the submitted work. Montrucchio, G declares personal fees for a lecture from Pfizer Gilead outside the submitted work. Murthy, S declares receiving salary support from the Health Research Foundation and Innovative Medicines Canada Chair in Pandemic Preparedness Research. Nichol, A. declares a grant from the Health Research Board of Ireland to support data collection in Ireland (CTN-2014-012), an unrestricted grant from BAXTER for the TAME trial kidney substudy, and consultancy fees paid to his institution from AM-PHARMA. Nseir S. declares lectures for Gilead, Pfizer, MSD, Biomérieux, Fischer and Paykel, and Bio-Rad, outside the submitted work. Openshaw, P. has served on scientific advisory boards for Janssen/J&J, Oxford Immunotech Ltd, GSK, Nestle, and Pfizer (fees to Imperial College). He is Imperial College lead investigator on EMINENT, a consortium funded by the MRC and GSK. He is a member of the RSV Consortium in Europe (RESCEU) and Inno4Vac, Innovative Medicines Initiatives (IMI) from the European Union. Peltan, I.D. declares grant support from the National Institutes of Health and, outside the submitted work, grant support from Centers for Disease Control and Prevention, National Institutes of Health, and Jannsen and payments to his institution from Regeneron and Asahi Kasei Pharma. Pesenti, A. declares personal fees from Maquet, Novalung/Xenios, Baxter, and Boehringer Ingelheim. Peytavin G. declares consulting fees (for lectures and/or participation in advisory boards) and travel grants from Gilead Sciences, Janssen, Merck, Takeda, Theratechnologies, and ViiV Healthcare. Poissy, J. declares personal fees from Gilead for lectures outside the submitting work. Povoa, P. declares personal fees (for lectures and advisory boards) from MSD, Technophage, Sanofi, and Gilead. Póvoas, D. declares consulting fees (for lectures and/or participation in advisory boards) from Roche and Viiv Healthcare; and travel/accommodation/meeting expenses from Abbvie, Gilead Sciences, Janssen Cilag, Merck Sharp & Dohme, and ViiV Healthcare. Rewa, O. declares honoraria from Baxter Healthcare Inc and Leading Biosciences Inc. Rössler, B. declares grants from CytoSorbent Inc. Rossanese, A. declares consulting fees (for lectures and/or participation to advisory boards) from Emergent BioSolutions and Sanofi Pasteur, but all outside of the frame of the submitted work. Săndulescu, O. has been an investigator in COVID-19 clinical trials by Algernon Pharmaceuticals, Atea Pharmaceuticals, Regeneron Pharmaceuticals, Diffusion Pharmaceuticals, and Celltrion, Inc. and Atriva Therapeutics, outside the scope of the submitted work. Semple, M.G. reports grants from DHSC National Institute of Health Research UK, from the Medical Research Council UK, and from the Health Protection Research Unit in Emerging & Zoonotic Infections, University of Liverpool, supporting the conduct of the study; other interest in Integrum Scientific LLC, Greensboro, NC, USA,  outside the submitted work. Serpa Neto, A. declares personal lecture fees from Drager outside the submitted work. Serrano-Balazote, P.. declares funding via his Institution from Novartis and Janssen, and personal fees or participation in advisory boards or participation to the speaker’s bureau of Roche, all outside of the submitted work. Shrapnel, S. participated as an investigator for an observational study analysing ICU patients with COVID-19 (for the Critical Care Consortium including ECMOCARD) funded by The Prince Charles Hospital Foundation during the conduct of this study. S. Shrapnel reports in-kind support from the Australian Research Council Centre of Excellence for Engineered Quantum Systems (CE170100009). Streinu-Cercel, Adrian has been an investigator in COVID-19 clinical trials by Algernon Pharmaceuticals, Atea Pharmaceuticals, Regeneron Pharmaceuticals, Diffusion Pharmaceuticals, and Celltrion, Inc., outside the scope of the submitted work. Streinu-Cercel, Anca has been an investigator in COVID-19 clinical trials by Algernon Pharmaceuticals, Atea Pharmaceuticals, Regeneron Pharmaceuticals, Diffusion Pharmaceuticals, and Celltrion Inc. and Atriva Therapeutics, outside the scope of the submitted work. Summers, C. reports that she has received fees for consultancy for Abbvie and Roche relating to COVID-19 therapeutics. She was also the UK Chief Investigator of a GlaxoSmithKline plc sponsored study of therapy for COVID and is a member of the UK COVID Therapeutic Advisory Panel (UK-CTAP). Outside the scope of this work, Dr. Summers’ institution receives research grants from the Wellcome Trust, UKRI/MRC, National Institute for Health Research (NIHR), GlaxoSmithKline, and AstraZeneca to support research in her laboratory. Susanne Dudman reports grants from Research Council of Norway grant no 312780. Tedder, R. reports grants from MRC/UKRI during the conduct of the study. In addition, R. Tedder has a patent United Kingdom Patent Application No. 2014047.1 “SARS-CoV-2 antibody detection assay” issued. Terzi, N. reports personal fees from Pfizer outside the submitted work. Timsit, J.F. participated in an advisory board for MSD, Pfizer, nabriva, Gilead, Shionoghi, Medimune outside the submitted work. JF Timsit declared lecture fees from MSD, Biomerieux, Pfizer, Shionoghi. Turtle, L. reports grants from MRC/UKRI during the conduct of the study and fees from Eisai for delivering a lecture related to COVID-19 and cancer, paid to the University of Liverpool. Ullrich, R. reports grant funding to his institution from Apeptico, APEIRON, Biotest, Bayer, CCORE, and Philips and personal fees from Biotest. He holds European patent EP15189777.4 “Blood purification device” and equity in CCORE Technology GesmbH, medical device research, and development company. Visseaux B. declares personal fees from BioMérieux, Qiagen, and Gilead and research grants from Qiagen, all outside the submitted work. West, E. reports grant funding from the Firland Foundation, the US CDC, and the Bill and Melinda Gates Foundation for studies of COVID-19, and grant funding from the US NIH for studies of other respiratory infections. Couffin-Cadiergues S. has nothing to declare relating to the present work. Søraas, A. Is a founder of the company Age Labs, which develops epigenetic tests, including COVID-19 severity.
